# Cognitive and Behavioral Factors Predicting the Decision to Vaccinate against COVID-19 in Clinical Psychiatric Population—A Cross-Sectional Survey

**DOI:** 10.3390/vaccines11020441

**Published:** 2023-02-15

**Authors:** Gabriela Mariana Marcu, Ana Maria Radu, Mihaela Dana Bucuță, Radu Sorin Fleacă, Ciprian Tanasescu, Mihai Dan Roman, Adrian Boicean, Ciprian Ionuț Bacilă

**Affiliations:** 1 Department of Psychology, Faculty of Social Sciences and Humanities, “Lucian Blaga” University of Sibiu, 550201 Sibiu, Romania; 2 Collective of Scientific Research in Neurosciences of the Clinical Psychiatry Hospital “Dr. Gheorghe Preda”, 550082 Sibiu, Romania; 3 Division of Physiology and Neuroscience, “Carol Davila” University of Medicine and Pharmacy, 020021 Bucharest, Romania; 4 Psychology Department, Faculty of Sociology and Psychology, West University of Timișoara, 300223 Timișoara, Romania; 5 Faculty of Medicine, “Lucian Blaga” University of Sibiu, 550169 Sibiu, Romania

**Keywords:** vaccination, COVID-19, decision-making, risk perception, trust, preventive behavior, prediction, psychiatric population, health beliefs

## Abstract

The spread of the COVID-19 virus created more than a medical crisis, while it also negatively affected the mental health of the general population. This context increased the vulnerability of the psychiatric population. While research interest highly targeted vaccine hesitancy and acceptance, many studies focused on trust issues—both in vaccine efficacy and in communication with authorities. Less is known about the psychological underpinnings of the COVID vaccination decision, specifically in the high-uncertainty circumstances due to the novelty of the virus. In a cross-sectional study, we investigated the predictive value of several cognitive (perceived risk, vulnerability, uncertainty, and trust in one’s decision) and behavioral (previous vaccinations, social media use, and practicing preventive behavior) factors, for the vaccination decision against COVID-19, for 252 psychiatric inpatients (data collected between September 2021 and February 2022). Demographics and diagnostics were also considered. We found a significant relationship between the “Perceived risk of vaccination” and the choice of vaccination (*χ*^2^(2, N = 252) = 58.59, *p* ≤ 0.001), and between the “Trust in own decision to vaccinate” and the decision to vaccinate (*χ*^2^(2, N = 252) = 31,5, *p* ≤ 0.001). The overall regression model was statistically significant (*χ*^2^ (9, N = 252) = 97.1, *p* < 0.001), with between 30% and 45% of the variance in the odds of a positive decision explained by the predictor set. The model coefficients analysis showed that an individual with a psychiatric disorder but with higher confidence in their decision had significant (*p* < 0.001) increased odds of the decision to vaccinate against COVID-19 by 893%. A former voluntary vaccination did not significantly associate with the decision to vaccinate against COVID-19 (*χ*^2^(1, N = 252) = 2.74, *p* > 0.05) in this special population. No other behavioral factors, diagnosis, or demographics were significant as predictors, for the clinical psychiatric population surveyed, except the educational level. Implications for future vaccination acceptance of this special population are discussed.

## 1. Introduction

The decision to vaccinate against COVID-19 has been a global research topic in the last three years, and the challenge to find its main triggers is even larger when working with psychiatric populations, where the decision-making process might be impaired. On the other side, people with mental health problems are considered vulnerable to epidemic outbursts. Together, context (mental health and epidemic) and individual factors might reveal a specific configuration that should be specifically addressed in increasing the vaccination rate.

This study focuses on the Romanian population and proposes a possible explanatory model for the vaccination decision of people with psychiatric disorders. The model is built based on the analysis of five factors, supposedly associated with the decision to administer the COVID-19 vaccine. The proposed factors are risk perception regarding the illness, risk perception of vaccination, perception of personal vulnerability, attitude toward uncertainty, and trust in personal choice. First, this paper briefly presents data from previous studies to bring supportive evidence for illustrating that the psychiatric population is a risk category for COVID-19 infection and, therefore, it could be vital to discover the factors that influence their decision-making process regarding vaccination. Secondly, we address the process of decision-making under uncertainty. Confidence in the healthcare system represents another factor that has a high impact on people’s decision to get vaccinated. In this study, we investigate variables related to or highly influential on an individual’s confidence in the healthcare system: the severity of the diagnosis and the presence of comorbidities, former voluntary vaccination, the use of social media, and the confidence in its contents, and practicing preventive behavior to protect against infection. (When discussing preventive behaviors, we translate that people engage in activities such as respecting social distance measures, wearing a face mask, regular ventilation of indoor spaces, quarantining, hand washing for at least 30 s, covering coughs and sneezes). Thirdly, we explore the psychological factors involved in the decision-making process in a medical context based on the Health Belief Model (HBM) [1] and the arguments for the vaccination against COVID-19, to illustrate reasons why it creates insecurity. The five factors included in the HBM model (perceived susceptibility, perceived severity, health motivation, perceived benefits, and perceived risks) as well as the impact of psychological characteristics on the vaccination decision or hesitancy are analyzed.

### 1.1. Psychiatric Population as a Highly Vulnerable Category for COVID-19 Infection

The COVID-19 pandemic has been a health emergency, which reached a pandemic in March 2020. The World Health Organization presents anti-COVID-19 vaccination as being critical to the end of this pandemic, but in various countries, such as it is in Romania, the vaccination rates remained low [2].

Furthermore, research shows that the pandemic context can significantly deteriorate individuals’ wellness and increase mental health issues. The stress caused by this medical crisis leaves the general population in distress, reduces life satisfaction, and even triggers reactive psychosis [3,4]. Therefore, the clinical psychiatric population, which is already a group characterized by vulnerability, would be constantly at considerable risk of rapid deterioration. For this reason, it is vital to understand the cognitive and behavioral factors that predict the intention to vaccinate against COVID-19 in individuals with clinical psychiatric issues.

Research suggests that patients with severe mental illness represent a high-risk category for developing an aggravated form of respiratory syndrome coronavirus [5], or a higher risk for COVID-19-related infection, hospitalization, and mortality [6,7,8,9]. A connection between having a psychiatric illness and a high risk for COVID-19-related negative outcomes [9] or developing more serious forms of the respiratory syndrome [6] was found, compared to the general population. Increased risk of COVID-19 infection was reported on participants with psychiatric disorders, within a sample of 421,014 participants from the UK Biobank. A total of 69.2% of individuals positively tested for the COVID-19 virus or having a cause of death related to the virus were individuals with a psychiatric diagnosis [9]. However, some evidence revealed no connection between increased COVID-19-related mortality and two of the mental health conditions (mood and anxiety disorders) [10]. One convergent point is that the psychiatric population has a two-to-three-times-higher mortality rate compared to the rest of the population [11,12], which also increases the risk of negative outcomes related to COVID-19 [13].

Moreover, mental illness was commonly associated with poor decision-making, such as non-compliance to treatment or suicide attempts [14,15]. Nevertheless, the decision-making deficits differ according to the diagnosis and associated symptoms. For example, anxiety is associated with two biases that impact the decision-making process—a response to threat-related information (response to threat-related information) and a tendency to interpret information gathered from ambiguous or neutral stimuli as negative (bias toward negative interpretation), which can lead to decision-making impairments [15]. Furthermore, a large body of literature brings evidence to the idea that cognitive dysfunctions in schizophrenia can occur even if the patient has proper symptom control [16], negatively altering the patient’s decision-making process [15]. The connection between mental illness and the impaired decision-making process is also salient in addictions, where inter-temporal choice is highly affected in addicted patients compared to healthy individuals [14].

### 1.2. Heterogeneity of Vaccination Determinants

Attempts to find an explanatory model of vaccination hesitancy/acceptance have led to heterogeneous results in the literature. Social, cognitive, behavioral, or political factors have been analyzed in attempts to identify the reasons that lead to the increase in vaccination acceptance. Conceptualizing the decision to vaccinate as a choice in a context of uncertainty and integrating this choice into a model of health beliefs, we can separate some influential factors that deserve further investigation:

#### 1.2.1. Cognitive Factors: Risk Perception and Uncertainty in Health Decisions

Risky decisions or choices between options of different probabilities of risk are often performed under the “bounded rationality” [17] condition. As a result, predictions for such choices must be investigated empirically. The effect of perceived risk in decisions was identified a long time ago in a version of a study on overestimating the results obtained in small samples, known as the “law of small numbers” [18]. Nowadays, the tendency extends to several situations in which people have erroneous intuitions about probabilities. This trend is amplified by the perception of risk.

Existing studies suggest that disease risk perception is a critical determinant of active and protective health behavior, the higher perception of risk being a predictor for the practice of general preventive behavior [19,20], or particularly as social distancing, but not hand washing [21]. However, the nature of the association between high-risk perception and health behavior also depends on the type of risk perception (deliberative, affective, experiential, etc.) and the accuracy of these perceptions [22]. In addition, it is speculated that people support emotional and experiential risk judgments (such as in anxiety and worry conditions) with greater conviction than deliberative, rational ones [23]. We tried to identify in this study which of the proposed risks could have such a similar valence, to influence the final vaccination decision.

However, the perceived risk per se is not sufficient to cause major changes in intentions and behavior, as evidenced by several studies on vaccination behavior [24], preventive behaviors in the COVID-19 pandemic [25], or intentions and behaviors in general [26]. Many of the studies on active or protective behaviors regarding illness used the “Health Belief Model” [1], which associates, in a rational paradigm, the increase in commitment to such behavior as the perceived risk of disease increases. Perceptions of disease risk, however, show significant variability, moderated by the degree of trust people have in their risk judgments or “belief in risk perception” [27]—the belief that this belief is correct. The model proposed by Taber and Klein suggests that the more confident people are that the threat is real, the more willing they are to engage in protective behavior that involves various costs (personal, material, etc.). As a result, to increase the accuracy of the predictions related to the vaccination decision, the level of perceived risk in the language is also important to determine the confidence that people have in their convictions, implicitly in those of risk estimation.

Interestingly, the lockdown has somewhat changed the weight of predictors in the vaccination decision, as the intention to vaccinate was found to be less affected by vaccination beliefs, while the perception of risk was increased during lockdown periods, with more people willing to be vaccinated against COVID-19 [28]. We could assume that the context of uncertainty was changed by the lockdown, which established a secure framework for perceiving a high risk [29].

Taber and Klein’s review concludes with only modest correlations between risk perception and people’s intentions/behavior, possibly due to a risk measuring problem [27]. Meanwhile, at the applicative level, interventions are still being designed in the hope that an increase in risk perception would lead to a change in behavior—initiatives that are often proved ineffective. Through this study, we want to provide a measure of association between the perceived risk (of getting the illness, of vaccination) and the decision to vaccinate, to establish the predictive value of risk perception for active COVID-19-related behaviors.

#### 1.2.2. Behavioral Factors: Vaccination History, Social Media Use, and Preventive Behaviors

Former voluntary vaccination has also been proposed in the current study to be associated with the acceptance of vaccination and as a factor to influence the process of decision-making regarding future vaccinations. Having a vaccination history could increase the acceptance rate of a COVID-19 vaccine by three times [28] or might raise the percentage of a new vaccine acceptance in 91.3% of the formerly vaccinated population [30]. Moreover, in a rapid systematic review [31] regarding the receptivity of the COVID-19 vaccine, the vaccination history was found as a strong predictor in six studies that associated the previous vaccination with the decision to get vaccinated [31]. There is also research on the vaccination for influenza that supports these findings, showing that the majority of individuals that chose to vaccinate once against influenza are most likely to get vaccinated in the next season as well [32].

The rapid opportunities for communication on social media platforms and the lack of scientific vetting create an environment where individual opinions, scientific data, or evidence are constantly debated and modified by people all around the world. If there is internet access, social media can reach significant audiences and any topic can be debated or searched for. In addition, it offers its users the possibility to reject the content they are not comfortable with and to subscribe to self-chosen streams of content that is in accordance with their personal views [33,34]. In theory, content can be transferred between users with different opinions, but this hardly occurs [35]. As social media offers an opportunity to access information quickly and effortlessly, many people look for advice or answers to various uncertainties on those platforms, including health-related information. Furthermore, there is a growing community of anti-vaccine groups that use social media networks to coordinate and manipulate discussions. A review of health-related misinformation on social media [36] shows that those groups receive significant attention and suggest that they have a significant influence on people’s decisions and behaviors. For example, out of 87 YouTube video clips on the topics “vaccine safety” and “vaccines and children”, 65% of them encouraged an anti-vaccine attitude, of which 36.8% did not offer empirical or scientific evidence. The misleading information from those clips receives a lot of popularity and viewers do not question their authenticity—hence the substantial number of trending video clips that have no scientific base [36].

Social media was also found to positively influence individuals to embrace preventive behaviors and to make them more prone to getting vaccinated. A study (N = 886) that examined the connection between social media exposure, preventive attitudes and behaviors, and risk perceptions of the COVID-19 virus found that high exposure to social media was associated with a higher risk perception, which was further connected to engagement in preventive behaviors and vaccination acceptance [37]. Positive associations were confirmed in a study regarding vaccination intentions in China, where a high engagement with COVID-19 content on social media was found positively correlated with vaccination acceptance [38].

Engagement in preventive behaviors was found to be associated with intentions to get vaccinated in several research studies, such as in a study from the USA (N = 592) where individuals without an intention to vaccinate have expressed lower levels of engagement in preventive behaviors such as avoiding social gatherings, wearing a mask, and frequently washing hands [39]. This is further supported by a study conducted in China, which showed similarly high percentages of protective behaviors such as constantly sanitizing hands, avoiding attending social gatherings, or wearing a facemask, which was positively associated with higher rates of vaccination intentions [38].

### 1.3. Current Study

The present study aims to explore the measure of association between cognitive and behavioral factors—such as perceived risk or vulnerability, confidence in one’s own decision, or previous preventive behaviors—and the choice for vaccination, in order to establish the predictive value of such factors in initiating a behavioral change in the health domain and on the decision to vaccinate.

Based on the existing evidence, we propose a prediction model, for the psychiatric population, with the following hypotheses:

When perceived risk of illness is high, compared with low, it will have a positive impact on the decision to vaccinate against COVID-19.

When perceived risk of vaccination is high, compared with low, it will have a negative impact on the decision to vaccinate against COVID-19.

When perceived personal vulnerability is high, compared with low, it will have a negative impact on the decision to vaccinate against COVID-19.

When perceived uncertainty is high compared with low, it will have a negative impact on the decision to vaccinate against COVID-19.

When trust in one’s decision to vaccinate is high compared to low, it will have a positive impact on the decision to vaccinate against COVID-19.

Former voluntary vaccination is positively associated with the decision to vaccinate against COVID-19

The use of *social media* and the confidence in its content are negatively associated with the decision to vaccinate against COVID-19 

Practicing a preventive behavior to protect against infection will have a positive impact on the decision to vaccinate against COVID-19

Besides the above-proposed predictors, some other variables are investigated in relation to the decision to vaccinate against COVID-19 to add to the literature with possible new hypotheses for further investigation; thus, demographics, the severity of the diagnosis, and the presence of comorbidities are investigated for their effect on the decision for vaccination in the sense of reducing it.

#### Diagnosis Severity, Comorbidities, and Vaccination Decision

Multiple studies gathered evidence supporting the idea that the severity of the diagnosis and the presence of co-morbidities are factors that influence individuals to get vaccinated [40,41,42]. Research regarding influenza vaccination (flu vaccination) suggested that individuals who perceive the seriousness of the disease and its risks are more likely to vaccinate [40,41]. Recent research [42] found that people who self-reported a chronic illness are more inclined to accept an anti-COVID-19 vaccine compared to healthy individuals. The presence of comorbidities might represent a factor that positively influences the acceptance of vaccination [42]. Empirical evidence supported the relationship between acceptance of COVID-19 vaccination and perceived severity of the disease [43] and also between the existence of co-morbidities and decisions on vaccination against influenza [44,45].

But research does not show homogenous results on the association of illness severity and vaccination, reflecting the probable variability of the factors definition. Oppositely to the previously presented results, another study did not find a significant correlation between the severity of the disease and vaccination acceptance, concluding that perceived benefits from the vaccine and cue to action are the variables that positively influenced the decision to vaccinate [32]. However, their sample was small (N = 299) and the study targeted a specific population—Israeli nurses. Therefore, those findings might not be relevant to other groups (psychiatric population, for example) or the general population [32].

## 2. Materials and Methods

### 2.1. Participants

A total of 252 patients (63.5% females, mean age 56.6; SD 13.4) hospitalized in the Clinical Hospital of Psychiatry “Dr. Gh. Preda” from Sibiu, Romania, were recruited between September 2021 and February 2022. Sample size was determined by a priori calculation (with G-power program) for a research power of 95% and an effect size of 0.5 (Cohen’s d). A total of 51.6% of participants were high-school-level graduates, while 19.4% had higher academic studies. Most of the participants were diagnosed with affective disorders (66.4%), while 13.9% were diagnosed with psychotic disorders, and the rest were diagnosed with addictive disorders (8%), personality disorders (6.3%) and degenerative disorders (5.5%). A total of 150 participants (59.5%) were married.

### 2.2. Design and Procedure

After being assigned a GDPR code and signing the informed consent, patients completed a questionnaire structured on several dimensions related to decision-making; the completion of the questionnaire was carried out by operators (medical staff from the hospital) and was completed by patients from several departments of the hospital, with various pathologies. The study was previously approved by the Ethics Committee of the hospital. In addition, the research followed the STROBE checklist for reporting observational studies [46]. The checklist is available in the supplementary materials (Table S1).

### 2.3. Instruments and Measurements

The questionnaire was built in Romanian, based on the consultation of the available literature on vaccination in general and vaccination against COVID-19 in particular. The instrument consisted, in addition to demographic and medical history data, of 29 items, structured on 5 dimensions—related to factors identified in the literature as being associated with the decision to vaccinate. Factors were selected to provide a possible explanatory model for the decision to vaccinate of people with mental disorders: risk perception regarding the disease (1), risk perception regarding vaccination (2), perception of personal vulnerability (3), attitude toward uncertainty (4), and confidence in one’s own beliefs (5). An English translation of the questionnaire can be found in the Supplementary Material as Annex S1.

The underlying theory in the construction of the questionnaire was the Health Belief Model (HBM) [1], an often-used model in studies investigating health-related behaviors. The model suggests that, if a person perceives a threat to their health, they will be consequently oriented to action, and if its perceived benefits outweigh the perceived barriers, then they are likely to take the recommended preventive health action.

Risk perception on disease was assessed by 8 Likert-type scale items that investigated dimensions of perception toward both COVID-19 and similar but familiar diseases such as influenza. Questions assessed perceived susceptibility (“How likely do you think it is to get sick from…”), perceived severity (“How severe do you think is the disease generated by the influenza virus/SARS-CoV-2”), perceived anxiety (“How scared you feel about contacting the influenza virus/SARS-CoV-2”), and confidence in the effectiveness of the vaccine. Perception of the risk of vaccination was evaluated through 8 items of the 5-step Likert scale (total disagreement/agreement) that formulated beliefs and behaviors associated with pro-health behavior and vaccination. (For example: “I need more information than what exists now, to decide to vaccinate”).

Perception of personal vulnerability was assessed by 4 questions on a 10-point Likert scale (for example: “I think my chances of getting infected with COVID-19 are” non-existent (1)–extremely high (10)).

The attitude toward uncertainty was assessed through 4 Likert-scale items, which operationalized the concept through questions such as “It is difficult for me to make a decision when I am not sure of the outcome”.

Confidence in one’s own beliefs was assessed on a 10-point Likert scale, by formulating a single item, related to the decision to vaccinate: “How sure am I of the decision made regarding the vaccination against COVID-19”.

In addition to the 5 cognitive factors, the questionnaire included items that measured behaviors hypothetically associated with pro-health attitudes: the use of social media, the choice and frequency of preventive behaviors (for example: wearing a mask, washing hands, avoiding crowded places), as well as a self-report of reasons for non-vaccination (such as “I don’t trust the effectiveness of the vaccine, I have other diseases and I’m afraid that they won’t get worse as a result of the vaccination, I don’t like having something imposed on me, I think the vaccine is bad for me, I read information that convinced me not to do it”).

**Figure 1:**
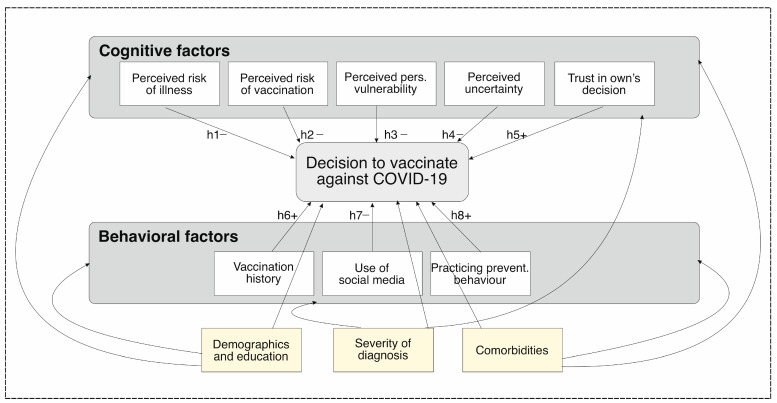
Schematic overview of the study’s conception. (Hypotheses are noted with “h”).

Demographic measures included: age, gender, studies, marital status, and number of children. Medical information contained the clinical diagnosis, time in hospital, treatment taken, comorbidities, smoking, and other vaccines taken (Figure 1).

### 2.4. Data Analysis

The Jamovi software (version 2.3.18.0, The Jamovi project, 2022, Sydney, Australia) was used for statistical analysis of the data. Descriptive analysis and normality tests were performed for demographics and predictor variables and frequency tables were drawn. For the dichotomous variables, the analysis was performed with contingency tables (association or incidence) and, later, the *χ*^2^ test was applied to explore the association between pairs of variables of interest. For all data, the threshold of significance was defined for the value of *p* < 0.05 for a confidence interval of 95%. For all tests, a “two-tailed” significance criterion of 5% was applied. 

The cognitive and behavioral factors were operationalized by outlined sections in the questionnaire, with several items for each section and each item counting for a specific variable/measure of the factor. Furthermore, six predictive factors to include in the analysis model were computed as means of specific variables’ raw scores, resulting in six computed continuous variables: “perceived risk” of illness/disease ((1) PERC_RISK_ILL) and of vaccination ((1) PERC_RISK_VACC), “Perceived personal vulnerability” ((2) PERC_VULN), “Perceived uncertainty” ((3) PERC_UNCERTAIN), “Use of social media” ((6) SOCIAL_MEDIA), and “Practicing a preventive behavior” ((7) PREV_BEHAV). The other two predictive factors were one categorial variable—“trust in one’s belief” to vaccinate ((4) TRUST_VACC_DEC, with 3 levels: low, moderate, and high)—and a dummy variable, “Former voluntary vaccination” ((5) PREV_VACC: with yes/no values). A binary logistic regression with a prediction model was conducted with these cognitive and behavioral factors as predictors. 

Bivariate association hypothesis testing was performed using the Pearson *χ*^2^ test for categorical (nominal) variables and Jamovi’s one-way ANOVA for non-parametric data for continuous variables with a nonnormal distribution. One analysis decision for avoiding confirmation bias was to test both for difference and equivalence. Running both tests for relevance and for equivalence supported the drawing of conclusions accordingly [47]. Thus, for all the tested effects that showed significance, equivalence tests (TOST procedure, Jamovi) were used, to conclude if the found effects were large enough to be worth further examination. The TOST procedure requires setting an upper and lower equivalence bound, which can be determined by using the smallest effect size of interest. The values that remain in between the thresholds are alleged to be equivalent to the lack of an effect that is worth analysis [48].

## 3. Results

### 3.1. Demographic Data

Among the 252 participants, 68.3% were vaccinated against COVID-19. Most participants were females (N = 160), out of whom 107 were vaccinated and 53 not vaccinated. Therefore, the vaccination rate among females was 66.87%. The male participants (N = 92) had a slightly higher vaccination rate, 70.65% (65 vaccinated participants and 27 not vaccinated). A characteristic associated with higher vaccination rates was the education level. A total of 71.4% of participants with higher education (N = 49) were vaccinated and 78.4% of subjects with secondary school studies (N = 130) while only 47.9% of individuals with primary studies (N = 73) were vaccinated. Other analyzed sociodemographic characteristics were age categories (18–39, 40–46, 65 and over), psychiatric diagnosis (affective disorders, psychotic disorders, addictive disorders, personality disorders, and degenerative disorders), smoking behavior, marital status, and previous voluntary vaccination. The frequency tables for each characteristic can be found in the supplementary materials (Table S2). 

Furthermore, the crosstabulation was performed to test the association of demographics and decision to vaccinate against COVID-19. As it can be seen in the frequencies (Table 1), a significant relationship was found only between the level of education and choice of vaccination (*χ*^2^ (dF = 4, N = 252) = 20.37, *p* < 0.01).

### 3.2. Factors Associated with the Vaccination Decision

For the variables hypothesized to predict the decision to vaccinate, we first performed a normality test. The distributions were significantly nonnormal for the variables: (1) perception of risk of vaccination (PERC_RISK_VACC) (W = 0.90, *p* < 0.01), (3) perceived uncertainty (PERC_UNCERTAIN) (W = 0.813, *p* < 0.01), (4) trust in one’s own decision (TRUST_OWN_DEC) (W = 0.732, *p* < 0.01), (6) social media behavior (SOCIAL_MEDIA_BEHAV) (W = 0.691, *p* < 0.01), and (7) preventive behavior (PREV_BEHAV) (W = 0.971, *p* < 0.01) according to Shapiro–Wilk tests (plots available in Supplementary materials, Table S2). The complete results can be seen in Table 2. Based on this outcome, a non-parametric test was used further.

**Figure 2:**
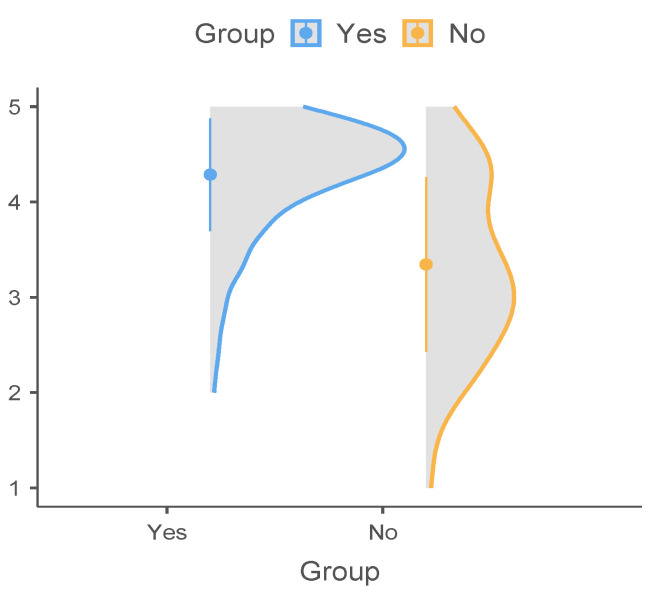
Decision to vaccinate in association with the perception of risk to vaccinate. The horizontal axis: groups of vaccinated/not vaccinated; the vertical axis: risk perception on vaccination; Higher numbers correspond to lower risk (e.g., 5 means lowest perceived risk and 1 means highest perceived risk).

A Chi-Square Test of Independence was performed to assess the relationship between the proposed cognitive and behavioral factors and the decision to vaccinate. There was a significant relationship between the “Perceived risk of vaccination” and the choice of vaccination (*χ*^2^(2, N = 252) = 58,59, *p* ≤ 0.001) (Table 3). Psychiatric patients with a high perceived risk of vaccination were less likely to decide to get a vaccine against COVID-19 compared to those with a low perceived risk (Figure 2). The TOST equivalence test was performed for the statistically significant difference and the null equivalence hypothesis was confirmed. The difference between groups fell outside the interval indicated by the established equivalence bounds (−0.5–0.5), which means that the found differences were not statistically equivalent (Table 4).

We also found a significant relationship between the “Trust in own decision to vaccinate” and the decision to vaccinate (*χ*^2^(2, N = 252) = 30.43, *p* ≤ 0.001). Psychiatric patients with a reduced trust in their decision to vaccinate were less likely to decide to get a vaccine against COVID-19 compared to those with a trust rated high (Table 5).

**Figure 3:**
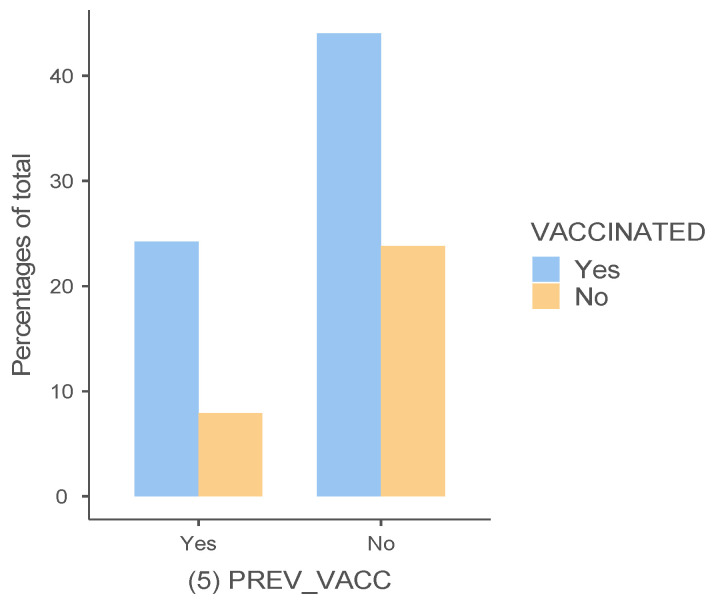
Previous voluntary vaccination and decision to vaccinate against COVID-19. The horizontal axis: groups of previously vaccinated/not vaccinated; the vertical axis: percentage of vaccinated against COVID-19.

Surprisingly, a former voluntary vaccination did not significantly associate with the decision to vaccinate against COVID-19 (*χ*^2^(1, N = 252) = 2.74, *p* > 0.05) (Table 6). Also, Figure 3. illustrates a comparison between previously vaccinated participants who also agreed to vaccinate against COVID-19 and participants who were not previously vaccinated but agreed to the immunization against COVID-19. 

For the factors expressed by continuous variables, Spearman’s rank correlations were computed to assess the relationship. There were highly significant positive correlations between perception of vulnerability (PERC_VULN) and perception of risk of illness (PERC_RISK_ILL), r(250) = 0.48, *p* < 0.001; (PERC_VULN) and perception of risk of vaccination (PERC_RISK_VACC), r(250) = 0.21, *p* < 0.001; preventive behavior (PREV_BEHAV) and PERC_RISK_VACC, r(250) = 0.25, *p* < 0.001. Negative significant correlations were between social media (SOCIAL_MEDIA) and PERC_RISK_ILL, r(250) = −0.12, *p* = 0.044; PERC_RISK_VACC, r(250) = −0.19, *p* = 0.002; PERC_VULN, r(250) = −0.17, *p* = 0.005. The complete correlation matrix can be found in Table 7. 

A TOST equivalence test was applied for the found significant correlations to test if the observed difference falls outside the prior specified equivalence bounds of −0.03 and 0.03. The conclusion of statistical equivalence was rejected for the pairs (1) PERC_RISK_VACC and (7) PREV_BEHA, 90%CI [0.157, 0.356]; (2) PERC_VULN and (1) PERC_RISK_VACC, 90%CI (0.107, 0.3113]; (2) PERC_VULN and (1) PERC_RISK_Illness, 90%CI [0.402, 0.565]. With 252 participants, the study had a 90% power to detect a correlation of 0.3 for this pair of variables.

Furthermore, we wanted to see which specific preventive behavior mostly correlates with the risk perception of vaccination, and the correlation matrix revealed that the chemicals (whether hand gel r(252) = 0.214, *p* < 0.001 or disinfectant r(252) = 0.218, *p* < 0.001) were the preferred preventive means for individuals with higher risk perception (Table 8).

### 3.3. Regression Model for Prediction of Decision to Vaccinate

A binary logistic regression was conducted to determine whether a psychiatric patient’s decision to vaccinate against COVID-19 could be predicted by the four cognitive and three behavioral factors. The overall model was statistically significant (*χ*^2^ (9, N = 252) = 97.2, *p* < 0.001) with between 30% and 45% of the variance in the odds of a positive decision explained by the predictor set (Table 9). Across both outcome categories, 81.7% of cases were accurately classified, with the specificity higher than sensitivity. The decision to vaccinate was correctly predicted in 82% of cases compared to 81.3% of no vaccination choice. The behavioral factor of practicing a preventing behavior was associated with an increase in the likelihood of a positive decision for vaccination against COVID-19 (OR = 1.677, 95%CI [0.9, 2.9]). The model coefficients analysis showed that an individual practicing preventive behavior has marginally significant (*p* = 0.058) increased odds of the decision to vaccinate against COVID-19 by 67.7%. In addition, an individual with higher confidence in their decision to vaccinate has significantly (*p* < 0.001) increased odds (OR = 8.936, 95%CI [2.6, 30]) to vaccinate, by 893%, than one with low confidence. Another predictor significantly related to the odds was the “Perceived risk of vaccination”, but the effect size (0.21) was small.

Tests to see if the data met the assumption of collinearity indicated that multicollinearity was not a concern (tolerance between 0.807 and 0.986, VIF between 1.01 and 1.24).

ROC curve analysis showed an optimal cut-off value of 0.3 (Sensitivity = 81.3%; Specificity = 82%). The predictive value of the regression model was 81.7%. (More graphs and tables in Supplementary Materials, Tables S3 and S4).

## 4. Discussion

The study’s main finding is the identification of psychiatric population characteristics that lower the odds of getting a vaccine against COVID-19: patients with lower education (elementary), who perceive a high risk of getting vaccinated and have little trust in their own decision on vaccination, and who practice little preventive behavior. No significant differences were found in sex, age, smoking behavior, marital status, and psychiatric diagnosis, regarding the decision to vaccinate against COVID-19, besides previous findings that linked higher risk perception with female gender, advanced age, poor health, city residence [20], low income [49], and reduced ability to calculate [50].

### 4.1. Psychiatric Patients vs. Normal Population in the Decision to Vaccinate against COVID-19

Even though many studies have already focused on vaccine hesitancy and decision factors for the normal population, little is presently known about the specificity of the decision to vaccinate in vulnerable populations such as psychiatric patients. The psychiatric population represents vulnerable groups in society, and it is essential to identify and understand the predictive factors that influence their vaccination decision about COVID-19, to be able to improve the uptake and consequently reduce their vulnerabilities [8,9]. The current study reveals the detriments behind the vaccination decision of psychiatric patients and the demographic and psychological predictive variables for vaccination against COVID-19. Surprisingly, the only demographic characteristic that predicts the vaccination decision for this population is the education level. Age did not induce statistically significant results, but we remark that the group aged between 40- and 46- years-old had the higher vaccination rate, in the psychiatric population, while participants in the age group over 60-years-old were in the lowest range of vaccination.

Mental illness could alter the decision-making process [14,15,51]. However, each condition could have a different impact on decision-making ability. In depression, for instance, the process could be longer compared to healthy individuals. Similarly, in schizophrenia, it could be slow, but also, less flexible and risky [52]. We conducted a crosstabulation to test the association of five diagnoses (affective disorder, psychosis, dementia, addiction, and personality disorder) and the decision to vaccinate against COVID-19. Our analysis did not reveal any significant differences between the included illnesses in regard to the vaccination decision (*χ*^2^ (dF = 5, N = 238) = 3.12, *p* = 0.54). Those results can only support the idea that the influence of one’s diagnosis over the decision-making process can vary. 

### 4.2. Cognitive Predictors: Risk Perception

Risk perception is one of the psychological factors involved in the decision-making process based on the Health Belief Model (HBM) [1]. In the case of COVID-19 vaccination decisions, the whole context is new and uncertain. To make the best decision under uncertainty, people try to know as much as possible about the probabilities of the consequences of each option; thus, the strategy becomes irrational because people generally tend to overweight lower probabilities. Our study reveals that the perceived risk of vaccination could be one of those triggers to impact the decision to vaccinate, as it might be linked to uncertain outcomes and, thus, increase the hesitancy for vaccination. As experiential states such as anxiety and worry were considered affective variants of risk perception, in conditions of uncertainty, analogous to deliberative perception [23], in this study, the perceived risk of vaccination was such a variant that might have induced an affective component in the vaccination decision. This would be in line with the association of preventive behaviors with perceptions of affective risk found in a recent meta-analysis [53].

Risk perception and trust are cognitive factors that recurrently occur in studies explaining human decision-making. We tested risk perception as a critical determinant of active and protective health behavior and we found a significant relationship between the “Perceived risk of vaccination”, but not of “perceived risk of illness” (as forms of protective behavior), and the decision to vaccinate (*χ*^2^(2, N = 252) = 58.59, *p* ≤ 0.001), but the effect size is small. In addition, the risk perception on vaccination significantly correlated (Spearman’s rho = 0.26, *p* < 0.001) with some other preventive behaviors, which is in line with previous research findings [19,20]. More specifically, we remark here that among all preventive behaviors of choice (wearing a mask, washing hands for 20s, avoiding crowded places, etc.), the ones that correlated most with the perceived risk of vaccination were the behaviors involving disinfecting substances, such as hand gel use (Spearman’s rho = 0.214, *p* < 0.001) and disinfectant substances use (Spearman’s rho = 0.218, *p* < 0.001). We could not tell whether this preference is characteristic of the psychiatric population or whether it is more general in the healthy population; therefore, this remains a hypothesis to be tested in further research.

### 4.3. Cognitive Predictors: Perceived Personal Vulnerability, Perceived Uncertainty, and Trust in One’s Own Decision to Vaccinate

No significant association was found in the current study between the perception of personal vulnerability, perceived uncertainty, and vaccine acceptance. Perceived vulnerability and known as well as perceived susceptibility were proposed for analysis as being major components of threat perception in the Health Belief Model. The model suggests that the greater the perceived personal vulnerability, the greater the perceived threat, and the more likely a person will perform health-oriented behaviors such as immunization. Perceived vulnerability is also an important component of threat appraisal in Protection Motivation Theory [54,55], but, at the moment, there is no definitive measurement scale or strategy to operationalize it. However, Likert-type response scales are usually utilized for measuring the perceived likelihood a negative event will occur, which we also used in the present study. That was more like an absolute measure of perceived vulnerability, which might have the weakness of inducing a confounding with expectations or intentions and which might explain the lack of association with vaccination adherence.

We also did not find a significant association between perceived uncertainty and decision to vaccinate against COVID-19, in the current study, which is in line with similar research: perceptions of COVID-19 uncertainty were not associated with vaccine intentions or testing [56] and perceived uncertainty did not influence attitudes toward vaccination [57].

### 4.4. Behavioral Predictors: Former Voluntary Vaccination

Previous vaccination against influenza was one of the influencing factors found to increase the odds of COVID-19 vaccination [30]. It can be argued that individuals’ perception over the COVID-19 infection was associated with their flu perception and, consequently, with their attitude toward immunization. This association was influenced by mass media or politicians’ discourses [28]. In this study, the regression model coefficients analysis did not show a significant association between vaccination history and decision to vaccinate against COVID-19 (*χ*^2^(1, N = 252) = 2.74, *p* > 0.05) for the psychiatric population. However, we should take into consideration the fact that the data for the current study were collected in the context of a national lockdown. This context (declared emergency state) could contribute to a change in the perceived risk assumption and, therefore, the previous vaccination would not be as relevant in comparison to the increased perception of risk or personal vulnerability. Similarly, other scholars reveal that there can be significant changes in perception that can further be reflected into behaviors in the time frame pre- and post-lockdown [28]. In addition, the participants in this study represent a specific vulnerable group and one can assume that their decision-making process would differ from the general population—hence, previous vaccination might not represent a highly influencing factor for the vaccination decision.

### 4.5. Behavioral Predictors: Social Media Engagement

While there is some evidence regarding the influence of social media on the decision to vaccinate, to our knowledge, no studies reported its influence on the psychiatric population. On one hand, studies show that social media can discourage individuals from preventive behaviors, encourage conspiracy theories, and spread misinformation, which can negatively impact on the vaccination decision or health-related behaviors [36,58,59]. On the other hand, if social media content is positive, it may encourage individuals to vaccinate [38]. In addition, social media was associated with a higher risk perception for COVID-19, which was also a factor that increased the chances of vaccination [37]. The literature on vaccination debates points out that social media disengages its users from debates and reinforces their own beliefs by confining the content they receive following their previously held beliefs [35]. COVID-19 conspiracy theories and social media were also linked in studies that found a negative association between folding COVID-19 conspiracy beliefs and engagement in protective behaviors. Moreover, the use of social media for accessing COVID-19 information was found to negatively influence individuals’ protective behaviors [58]. For the current study, as informed by the cited literature, we expected social media use to positively influence individuals’ perspectives on vaccination and their engagement in health-protective behaviors.

The psychiatric population represents a vulnerable group, and their diagnosis could negatively influence their decision-making process [9,14,15]. Considering that those studies focused on the association between social media influence and vaccination decision had a sample of healthy individuals (without a psychiatric diagnosis), one can argue that psychiatric patients could be even more susceptible to social media’s influence regarding vaccination decisions. Nevertheless, the current research did not gather data about what type of content the participants accessed on social media, but only the quantitative time spent on social networks such as Facebook or Instagram. Our analysis revealed negative significant correlations between social media engagement and perceived risk of illness (r(250) = −0.12, *p* = 0.044), perceived risk of vaccination (r(250) = −0.19, *p* = 0.002), and perceived vulnerability (r(250) = −0.17, *p* = 0.005). These correlations can only reveal that there is an association between social media use and the other three potential predictors explored in this study.

One explanation for this result could be the positively skewed distribution of social media use in our studied population. More than 80% of the patients in the study used social media less than an hour a day, data that are not similar to the healthy population (the use of social media for the years 2020–2022, in the age range of 16–64 years, was an average of 2.5 h, worldwide) [60]. Therefore, this negative correlation we found in the study should be interpreted with caution, as more studies in psychiatric patients (perhaps with a less skewed social media use distribution curve) are needed to confirm an inverse relationship with risk perception. We cannot conclude that social media is a factor that decreases the odds of getting vaccinated for the psychiatric population, while, besides this correlation, no other significant findings support this idea.

### 4.6. Behavioral Predictors: Preventive Behaviors

Practicing preventive behavior correlates with confidence in one’s decision to vaccinate (Spearman’s rho = 0.207, *p* < 0.001), and again, the highest correlations are with the two behaviors involving disinfectants: hand gel use (Spearman’s rho = 0.210, *p* < 0.001) and use of other disinfectant substances (Spearman’s rho = 0.206, *p* < 0.001). The results are convergent with the Taber and Klein model [27], which suggests a positive association between people engaging in protective behavior and confidence in their belief about the threat. Individuals who engage in preventive behaviors could be more motivated, self-aware, or self-efficient regarding their health and, therefore, they would take measures to conserve their state of health—hence, they engage in preventive behaviors [49]. In addition, risk perception on vaccination significantly correlated (Spearman’s rho = 0.26, *p* < 0.001) with some preventive behaviors; the result is supported by findings from other studies [19,20]. Therefore, the association between risk perception and engagement in preventive behaviors could be explained as follows. If the perceived risk of a certain event is high, an individual would take action to protect themself against the situation associated with the high risk. However, the analyses conducted in this study cannot provide explanations for the individuals’ choices of preventive behaviors, such as reasons why they prefer to choose using disinfectants. 

### 4.7. Vaccine Coverage—Country-Specific Differences

The World Health Organization statistics on the anti-COVID-19 vaccination showed very low vaccination rates at the national level in Romania [2]. Even at the end of 2022, when most European countries reported vaccination rates of 80–99%, vaccine coverage in the Romanian general population was low at the country level (46.5%—as reported by the Ministry of Health and the European Center for Disease Prevention and Control (ECDC)) a phenomenon that deeply concerned the specialists in the field of public health. This was also a low coverage compared to the average (75.2%) found in 23 countries [61]. Regarding the rate per county, in Sibiu County where the study took place, the rate was not much different from the national value (41.45%). However, the vaccination percentage in psychiatric patients was 68.3% in the current study, showing a large difference compared to the national and county number. This difference is very specific to the Romanian population and contrasts with other reported coverage rates, such as those in a study on N = 1151 psychiatric patients in Belgium, where the willingness to receive a COVID-19 vaccination was found to be as high as in the general population [62] or a similar finding reported for the Danish study [63].

The large difference in the percentage of vaccination between the psychiatric population included in the study (68.3%) and the general population of Romania (40–45%) could be explained by the fact that the average age of the psychiatric population in the study is over 55 years, with most patients being included in a risk group with priority for vaccination, which was included in the first vaccination rounds when the anti-COVID-19 vaccine was available.

### 4.8. Psychiatric Population Peculiarities

There is one important thing to point out regarding the results of risk perception on vaccination of this special population. It is known that even for the healthy population, the perception of risk is not always clear and often cannot be strongly supported; one could wonder what the case in populations with mental health issues would be. When the perception of risk is unclear, it does not fully capture how people think about risk. Thus, those people who cannot formulate their own beliefs or cannot estimate whether they are correct are unlikely to form a perception of risk that is predictive of intentions and behaviors. We expected people with different psychiatric illnesses to show differences in risk perception, but that did not happen. This might be because perceptions of affective and experiential risk are less dependent on formulation and that can also explain why they are often stronger in predicting behavior than deliberate risk assessments [27].

### 4.9. Limitations

Even though low education levels were found to be associated with the prediction of a negative vaccination decision in this study, as in a recent Chinese study on vaccination coverage in psychiatric patients [64], the current study did not assess the psychological status of the patients. In addition, the Health Belief Model is not the only model that has proven useful in investigating the determinants of health-related behaviors, such as vaccination. Other studies have proposed other models, such as the COM-B model (ability, motivation, opportunity–behavior) [65] that might reveal other reliable factors of encouraging vaccination.

## 5. Conclusions

To conclude, the present study investigated the relationship between different cognitive and behavioral factors and the decision to vaccinate against COVID-19 on a specific vulnerable group: the psychiatric population. The study expanded the theoretical perspective in regard to the vaccination decision. There is considerable available literature concerning the vaccine hesitancy and trust issues; few studies examined the psychological factors involved in the decision-making process under uncertainty. Our study was conducted in a Romanian Psychiatric Hospital and gathered 252 psychiatric patients.

Despite some general expectations, such as having the clinical diagnosis or a previous vaccination as an influence on the decision to vaccinate, the current study’s results did not confirm these factors as reliable predictors but showed a relatively narrow configuration of factors to act as predictors. Among the seven factors considered, four cognitive factors (perceived risk, vulnerability, uncertainty, and trust in one’s decision) and three behavioral (previous vaccinations, social media use, and preventive behavior), only two revealed a significant predictive value. We found a significant relationship between the “Perceived risk of vaccination” and the choice of vaccination (*χ*^2^(2, N = 252) = 58.59, *p* ≤ 0.001), and between the “Trust in own decision to vaccinate” and the decision to vaccinate (*χ*^2^(2, N = 252) = 31.5, *p* ≤ 0.001). The model of prediction integrates the risk of vaccination and confidence in one’s choice as predictors, supported by the overall regression model.

Our results raise some points of interest in targeting possible hesitancy in vaccination decisions, which could help in directing future health strategies, for people with psychiatric disorders. The study findings show no specific determinant (either cognitive or behavioral) that weighs most in the decision to vaccinate against COVID-19, but some associated small influences that are worth further investigation in order to build on a strong motivational strategy. It is important that such factors could be addressed by therapeutic techniques (such as cognitive-behavioral, e.g., reappraisal, reframing, or contingency management) in order to increase the odds of vaccination, especially for highly vulnerable populations.

## Figures and Tables

**Table 1:** Sociodemographic characteristics and decision to vaccinate, in the psychiatric population.

Cross Table for Dependent: Vaccinated				
	N	Yes (N = 172)	No (N = 80)	Statistical Test
**SMOKER: No**	250	0.6 107/170 (62.94%)	0.7 57/80 (71.25%)	X_1_^2^ = 1.66, *p* = 0.202
**AGE**	252			X_2_^2^ = 5.85, *p* = 0.052
18–39 years		0.1 14/172 (8.14%)	0.2 14/80 (17.5%)	
40–64 years		0.6 107/172 (62.21%)	0.5 40/80 (50%)	
65 and above 65 years		0.3 51/172 (29.65%)	0.3 26/80 (32.5%)	
**SEX: Female**	252	0.6 107/172 (62.21%)	0.7 53/80 (66.25%)	X_1_^2^ = 0.38, *p* = 0.542
**DIAGNOSTIC**	238			X_4_^2^ = 3.12, *p* = 0.542
Affective disorder		0.7 111/160 (69.38%)	0.6 47/78 (60.28%)	
Psychosis		0.1 19/160 (11.88%)	0.2 14/78 (17.95%)	
Dementia		0.0 7/160 (4.38%)	0.1 6/78 (7.69%)	
Addiction		0.1 10/160 (6.25%)	0.1 6/78 (7.69%)	
Personality disorder		0.1 10/160 (6.25%)	0.1 5/78 (6.41%)	
**MARITAL STATUS: No**	152	0.4 68/172 (39.53%)	0.4 34/80 (42.5%)	X_1_^2^ = 0.20, *p* = 0.662
**STUDIES**	252			
Primary		0.2 35/172 (20.35%)	0.5 38/80 (47.5%)	
Secondary		0.6 102/172 (59.3%)	0.3 28/80 (35%)	
Higher		0.2 35/172 (20.35%)	0.2 14/80 (17.5%)	
**SOCIAL MEDIA**	252			X_4_^2^ = 0.94, *p* = 0.922
0 h		0.4 74/172 (43.02%)	0.4 30/80 (37.5%)	
Under 1 h		0.4 64/172 (37.21%)	0.4 32/80 (40%)	
1–3 h		0.2 31/172 (18.02%)	0.2 16/80 (20%)	
3–5 h		0.0 2/172 (1.16%)	0.0 1/80 (1.25%)	
Above 5 h		0.0 1/172 (0.58%)	0.0 1/80 (1.25%)	
**TRUST IN SOCIAL MEDIA**	165			X_2_^2^ = 0.18, *p* = 0.912
Low		0.6 67/108 (62.04%)	0.6 35/57 (61.4%)	
Medium		0.3 30/108 (27.78%)	0.3 15/57 (26.32%)	
High		0.1 11/108 (10.19%)	0.1 7/57 (12.28%)	

N is the number of non-missing values.

**Table 2:** Predictors normality test.

	N	Mean	Median	SD	Variance	Minimum	Maximum	W	*p*
Perceived risk of vaccination	252	3.987	4.286	0.832	0.693	1.00	5.00	0.900	<0.001 *
Perceived risk of illness	252	5.412	5.500	1.473	2.171	1.00	9.13	0.990	0.076 *
Perceived vulnerability	252	5.209	5.250	1.732	3.000	1.00	9.25	0.984	0.007 *
Perceived uncertainty	252	6.304	6.500	0.806	0.649	2.00	7.00	0.813	<0.001 *
Trust in own decision	252	8.190	10.000	2.566	6.585	1	10	0.732	<0.001 *
Social media engagement	252	0.992	0.500	1.404	1.970	0.00	10.50	0.691	<0.001 *
Preventive behavior	252	3.783	3.917	0.674	0.455	1.50	5.00	0.971	<0.001 *

* Shapiro–Wilk.

**Table 3:** Associations between cognitive and behavioral factors and decision to vaccinate.

Cognitive and Behavioral Factors	*χ* ^2^	df	*p*	ε^2^
Perceived risk of illness	6.8297	1	0.009	0.02721
Perceived risk of vaccination	58.5887	1	<0.001	0.23342
Perceived vulnerability	0.0584	1	0.809	2.33 × 10^−4^
Perceived uncertainty	1.6584	1	0.198	0.00661
Social media engagement	0.5210	1	0.470	0.00208
Preventive behavior	1.4279	1	0.232	0.00569

Cognitive factors: “perceived risk of illness/disease” and of vaccination, “Perceived personal vulnerability”, “Perceived uncertainty”. Behavioral factors: “Use of social media” and “Practicing a preventive behavior”.

**Table 4:** Equivalence test and effect size.

			90% Confidence Interval	
		Estimate	Lower	Upper
**(1) PERC_RISK_VACC**	Cohen’s d(av)	1.22	0.982	1.49
	Raw	0.941	0.756	1.13

Note. Denominator set to the average SD.

**Table 5:** Trust in one’s decision and decision to vaccinate against COVID-19.

Trust in One’s Decision to Vaccinate	VACCINATED		Total
	Yes	No	
**low**	5	16	21
Medium	26	23	49
High	141	41	182
**Total**	172	80	252
	Value	Df	*p*
***χ* ^2^**	31.5	2	<0.001

N = 252, Cognitive factor: “Trust in one’s belief” to vaccinate.

**Table 6:** Previous voluntary vaccination and decision to vaccinate against COVID-19.

		VACCINATED		Total
Previously Vaccinated		Yes	No	
Yes	Observed	61	20	81
	% Within row	75.3%	24.7%	100.0%
No	Observed	111	60	171
	% Within row	64.9%	35.1%	100.0%
Total	Observed	172	80	252
	% Within row	68.3 %	31.7%	100.0%
		Value	df	*p*
***χ* ^2^**		2.74	1	0.098

N = 252, Behavioral factor: “Former voluntary vaccination”.

**Table 7:** Correlation matrix of cognitive and behavioral factors.

		Perceived Risk of Illness	Perceived Risk of Vaccination	Perceived Vulnerability	Perceived Uncertainty	Social Media Engagement	Preventive Behavior
Perceived risk of illness	Spearman’s rho	—					
	*p*-value	—					
Perceived risk of vaccination	Spearman’s rho	−0.062	—				
	*p*-value	0.330	—				
Perceived vulnerability	Spearman’s rho	0.488 ***	0.211 ***	—			
	*p*-value	<0.001	<0.001	—			
Perceived uncertainty	Spearman’s rho	0.083	0.041	0.063	—		
	*p*-value	0.190	0.519	0.318	—		
Social media engagement	Spearman’s rho	−0.127 *	−0.196 **	−0.177 **	0.076	—	
	*p*-value	0.044	0.002	0.005	0.230	—	
Preventive behavior	Spearman’s rho	−0.039	0.259 ***	0.049	0.080	−0.014	—
	*p*-value	0.539	<0.001	0.439	0.203	0.823	—

* *p* < 0.05, ** *p* < 0.01, *** *p* < 0.001.

**Table 8:** Correlation matrix of preventive behaviors and risk perception about vaccination.

		Perceived Risk of Vaccination	Mask	Hand Gel	Washing 20s	Avoid Crowds	Disinfectants	Search Info
Perceived risk of vaccination	Spearman’s rho	—						
	*p*-value	—						
Mask	Spearman’s rho	0.002	—					
	*p*-value	0.975	—					
Hand Gel	Spearman’s rho	0.214 ***	0.414 ***	—				
	*p*-value	<0.001	<0.001	—				
Washing 20s	Spearman’s rho	0.163 *	0.447 ***	0.590 ***	—			
	*p*-value	0.010	<0.001	<0.001	—			
Avoid crowds	Spearman’s rho	0.152 *	0.339 ***	0.375 ***	0.526 ***	—		
	*p*-value	0.016	<0.001	<0.001	<0.001	—		
Disinfectants	Spearman’s rho	0.218 ***	0.138 *	0.459 ***	0.431 ***	0.432 ***	—	
	*p*-value	<0.001	0.032	<0.001	<0.001	<0.001	—	
Search info	Spearman’s rho	0.153 *	0.100	0.125	0.227 ***	0.308 ***	0.479 ***	—
	*p*-value	0.021	0.131	0.063	<0.001	<0.001	<0.001	—

* *p* < 0.05, *** *p* < 0.001.

**Table 9:** Regression model fit measures and coefficients.

Model Fit Measures								
						Overall Model Test		
Model	Deviance	AIC	R²_McF_	R²_CS_	R²_N_	*χ* ^2^	df	*p*
1	218	238	0.309	0.320	0.449	97.2	9	<0.001
**Model Coefficients—VACCINATED**								
						**95% Confidence Interval**		
**Predictor**	**Estimate**	**SE**	**Z**	***p***	**Odds Ratio**	**Lower**	**Upper**	
Intercept	0.6092	1.980	0.308	0.758	1.839	0.0379	89.105	
(5) PREV_VACC:								
No—Yes	0.3707	0.392	0.946	0.344	1.449	0.6722	3.123	
(4) TRUST_VACC_DEC:								
Low-high	2.1901	0.619	3.540	<0.001	8.936	2.6579	30.045	
Medium-high	1.0555	0.419	2.520	0.012	2.873	1.2645	6.529	
(1) PERC_RISK_ILL	0.2333	0.146	1.600	0.110	1.263	0.9489	1.680	
(1) PERC_RISK_VACC	−1.5639	0.239	−6.530	<0.001	0.209	0.1309	0.335	
(2) PERC_VULN	−0.0309	0.120	−0.257	0.797	0.970	0.7660	1.227	
(3) PERC_UNCERTAIN	0.1504	0.209	0.720	0.471	1.162	0.7719	1.750	
(6) SOCIAL_MEDIA	−0.0399	0.128	−0.312	0.755	0.961	0.7476	1.235	
(7) PREV_BEHAV	0.5170	0.273	1.895	0.058	1.677	0.9824	2.863	

Note. Estimates represent the log odds of “VACCINATED = No” vs. “VACCINATED = Yes”.

## Data Availability

The data presented in this study are available on request from the corresponding author.

## Supplementary Materials

The following supporting information can be downloaded at: https://www.mdpi.com/article/10.3390/vaccines11020441/s1, Table S1: SM_STROBE Checklist.pdf; Table S2: SM_Normality_tests.pdf; Table S3: SM_Logistic_Regression_results.pdf; Table S4: SM_TOST_correlations.pdf; Annex S1: SM_Annex S1—Questionnaire.pdf.
