# Supplementary material for: Cognitive and Behavioral Factors Predicting the Decision to Vaccinate against COVID-19 in Clinical Psychiatric Population—A Cross-Sectional Survey
Source: Vaccines (Basel). 2023 Feb 15;11(2):441. doi: 10.3390/vaccines11020441 (PMC9961952; doi:10.3390/vaccines11020441)
Supplement: Supplementary file 1 [file vaccines-11-00441-s001.zip › SM_Annex S1 - Questionnaire.pdf]

QUESTIONNAIRE DEC_VCC_01

HIPPA Code: ____
(the first two letters of the name, the first two letters of the surname, the first letter of the city, the last two digits of the CI)

Consent to participate in research

I voluntarily agree to participate to this study. I understand that even if now I agree to participate, I can withdraw at any time or refuse to answer to any question without a consequence of any kind.

The purpose and nature of this study was explained to me and I was given the opportunity to ask questions about the study. I understand that participation involves determining the acceptance and risk perception of the COVID-19 vaccine in Romania.

I understand that I will not directly benefit from participating in this research.

I understand that all information I provide for this study will be treated confidentially.

I understand that in any reporting of the research results my identity will remain anonymous.

By choosing yes, I agree to participate in this study

By choosing the NO option, I will not participate in this study

A. YES
B. NO

(I) PERSONAL / MEDICAL DATA

Age: __________________________________ Gender: ___________
City / County:____
Diagnosis at admission:
____
____

Length of hospitalization (days): ____________________
Other evident/reported co-morbidities:____
Treatment: ____
____
Smoking? Yes / No

Did he/ she do other types of vaccine? yes / no when_______
Did he/she have any problems after the vaccine? yes / no. How did they manifest?
____

©2021, Clinical Hospital of Psychiatry "Dr. Gheorghe Preda" Sibiu, "Lucian Blaga" University from Sibiu

Any other health issues that should be mentioned?

____

(II) PERCEIVED DISEASE RISK

1. How likely do you think you are to get the flu?

1	2	3	4	5	6	7	8	9	10
○	○	○	○	○	○	○	○	○	○

1 = it is not likely at all 10 = extremely likely

2. How likely do you think you are to get Covid-19?

1	2	3	4	5	6	7	8	9	10
○	○	○	○	○	○	○	○	○	○

1 = it is not likely at all 10 = extremely likely

3. How severe do you think the flu is?

1	2	3	4	5	6	7	8	9	10
○	○	○	○	○	○	○	○	○	○

1 = it is not likely at all 10 = extremely likely

4. How severe do you think the disease caused by the SARS-Cov-2 virus is?

1	2	3	4	5	6	7	8	9	10
○	○	○	○	○	○	○	○	○	○

1 = it is not likely at all 10 = extremely likely

5. How scared do you feel about catching the flu?

1	2	3	4	5	6	7	8	9	10
○	○	○	○	○	○	○	○	○	○

1 = I don't feel scared at all 10 = extremely scared

6. How scared do you feel about contacting the Sars-Cov-2 virus?

©2021, Clinical Hospital of Psychiatry "Dr. Gheorghe Preda" Sibiu, "Lucian Blaga" University from Sibiu

1	2	3	4	5	6	7	8	9	10
○	○	○	○	○	○	○	○	○	○

1 = I don't feel scared at all 10 = extremely scared

7. How confident are you in the effectiveness of the flu vaccine?

1	2	3	4	5	6	7	8	9	10
○	○	○	○	○	○	○	○	○	○

1= not confident 10 = extremely confident

8. How confident are you in the effectiveness of the Covid-19 vaccine?

1	2	3	4	5	6	7	8	9	10
○	○	○	○	○	○	○	○	○	○

1= not confident 10 = extremely confident

(III) PERCEPTION OF PERSONAL VULNERABILITY

1. Imagining myself infected with COVID-19 is:

1	2	3	4	5	6	7	8	9	10
○	○	○	○	○	○	○	○	○	○

1 = extremely easy 10 = extremely difficult

2. I am sure that I will not get infected with COVID-19:

1	2	3	4	5	6	7	8	9	10
○	○	○	○	○	○	○	○	○	○

1= not at all sure 10 = extremely sure

3. I feel vulnerable to COVID-19 infection:

©2021, Clinical Hospital of Psychiatry "Dr. Gheorghe Preda" Sibiu, "Lucian Blaga" University from Sibiu

1	2	3	4	5	6	7	8	9	10
○	○	○	○	○	○	○	○	○	○

1 = I do not feel vulnerable at all   10 = extremely vulnerable

4. I think my chances of getting infected with COVID-19 are:

1	2	3	4	5	6	7	8	9	10
○	○	○	○	○	○	○	○	○	○

1 = non-existent / zero   10 = extremely high

(IV) THE PERCEIVED RISK REGARDING VACCINATION

I agree with the statements below:

	**0**	**1**	**2**	**3**	**4**
	Do not agree at all	Rather disagree	Neither agree nor disagree	I rather disagree	Totally agree
1. Vaccines are effective in preventing disease					
2. Getting through the disease provides better immunity than the vaccine					
3. I usually do what my doctor recommends					
4. Vaccinating myself against Covid-19 is a good way to protect myself from the virus infection					
5. Family and friends generally think the vaccine is a good idea					
6. To protect public health we should follow the government's vaccine instructions					

©2021, Clinical Hospital of Psychiatry "Dr. Gheorghe Preda" Sibiu, "Lucian Blaga" University from Sibiu

7. Patients at risk should be vaccinated first					
8. I need more information than I have now to decide whether to get vaccinated					

(V) ATTITUDE TOWARDS UNCERTAINTY

For the statements below, mark the extent to which you agree or disagree:

1. There is always a right way and a wrong way to do things

totally agree | moderately agree | easily agree | neutral | slightly disagree | moderate disagree | totally diasagree

2. Practically, every problem also has a solution

totally agree | moderately agree | easily agree | neutral | slightly disagree | moderate disagree | totally diasagree

3. I feel relieved when an unclear situation becomes clear

totally agree | moderately agree | easily agree | neutral | slightly disagree | moderate disagree | totally diasagree

4. It is difficult for me to make a decision when I am not sure of the outcome

totally agree | moderately agree | easily agree | neutral | slightly disagree | moderate disagree | totally diasagree

(VI) OTHERS

Sociodemographic factors

- Married YES / NO
- Children: YES / NO, if yes, how many? ____, what age? _________________
- Last school graduated:

©2021, Clinical Hospital of Psychiatry "Dr. Gheorghe Preda" Sibiu, "Lucian Blaga" University from Sibiu

Use of social media

1. Social networks used:

□ Facebook: under 1h / day; 1-3 hours/day; 3-5 hours/day; over 5 hours/day
□ Instagram: under 1h / day; 1-3 hours/day; 3-5 hours/day; over 5 hours/day
□ TikTok: under 1h / day; 1-3 hours/day; 3-5 hours/day; over 5 hours/day
□ Tinder: under 1h / day; 1-3 hours/day; 3-5 hours/day; over 5 hours/day
□ Telegram: under 1h / day; 1-3 hours/day; 3-5 hours/day; over 5 hours/day
□ Others. Which?________________

2. I am confident about the information on social networks, regarding COVID-19:

1	2	3	4	5	6	7	8	9	10
○	○	○	○	○	○	○	○	○	○

1 = not at all sure 10 = extremely sure

Preventive behaviors adopted:

What methods have I used to protect myself from infection with COVID-19?

□ Wearing the mask very often / often / neither often nor rarely / rarely / very rarely
□ Use of disinfectant gel very often / often / neither often nor rarely / rarely / very rarely
□ Hand washing min 20 s very often / often / neither often nor rarely / rarely / very rarely
□ Avoiding crowded places very often / often / neither often nor rarely / rarely / very rarely
□ Use of disinfectants very often / often / neither often nor rarely / rarely / very rarely
□ Information from several sources very often / often / neither often nor rarely / rarely / very rarely
□ Others. Which? _____________ very often / often / neither often nor rarely / rarely / very rarely

Have you been vaccinated against COVID-19? YES/NO

If YES, month_____, year ______, which vaccine _____________, no doses__
If NO, what were the reasons?

□ I do not trust the effectiveness of the vaccine
□ I have other diseases and I am afraid that they will get worse after the vaccination
□ I do not like to be coerced
□ I think the vaccine is making me sick
□ I read information that convinced me not to do it (like? ____ ____
□ Others. Which? _____________

©2021, Clinical Hospital of Psychiatry "Dr. Gheorghe Preda" Sibiu, "Lucian Blaga" University from Sibiu

Confidence in your own beliefs

How confident am I about my decision to get vaccinated against COVID-19:

1	2	3	4	5	6	7	8	9	10
○	○	○	○	○	○	○	○	○	○

1 = not at all sure 10 = extremely sure

Date: ___________________________ Operator (initials) _________

Place/section: ____________

©2021, Clinical Hospital of Psychiatry "Dr. Gheorghe Preda" Sibiu, "Lucian Blaga" University from Sibiu
