# Supplementary material for: Cognitive and Behavioral Factors Predicting the Decision to Vaccinate against COVID-19 in Clinical Psychiatric Population—A Cross-Sectional Survey
Source: Vaccines (Basel). 2023 Feb 15;11(2):441. doi: 10.3390/vaccines11020441 (PMC9961952; doi:10.3390/vaccines11020441)
Supplement: Supplementary file 1 [file vaccines-11-00441-s001.zip › Table S1 SM_STROBE Checklist.pdf]

Table S1: STROBE Statement—checklist of items that should be included in reports of observational studies

	Item No.	Recommendation	Page No.	Relevant text from manuscript
**Title and abstract**	1	(*a*) Indicate the study's design with a commonly used term in the title or the abstract	1	Title & Abstract
		(*b*) Provide in the abstract an informative and balanced summary of what was done and what was found	1-2	Abstract
**Introduction**				
Background/rationale	2	Explain the scientific background and rationale for the investigation being reported	1-5	Introduction
Objectives	3	State specific objectives, including any prespecified hypotheses	5	Introduction sect 1.3
**Methods**				
Study design	4	Present key elements of study design early in the paper	6-7	Materials andMethods
Setting	5	Describe the setting, locations, and relevant dates, including periods of recruitment, exposure, follow-up, and data collection	6	Materials and Methods (paragraphs 3-4)
Participants	6	(*a*) *Cross-sectional study*—Eligibility criteria and the sources and methods of selection of participants.	6	Materials and Methods (paragraphs 3)
Variables	7	Clearly define all outcomes, exposures, predictors, potential confounders, and effect modifiers. Give diagnostic criteria, if applicable	6-7	Materials andMethods
Data sources/ measurement	8*	For each variable of interest, give sources of data and details of methods of assessment (measurement). Describe comparability of assessment methods if there is more than one group	8	Materials and Methods sect 2.3
Bias	9	Describe any efforts to address potential sources of bias	8	Materials and Methods: section 2.4 Data analysis
Study size	10	Explain how the study size was arrived at	6	Participants, 2.1 G-power software

Quantitative variables	11	Explain how quantitative variables were handled in the analyses. If applicable, describe which groupings were chosen and why	8	Materials and Methods: section 2.4 Data analysis (paragraph 2)
Statistical methods	12	(*a*) Describe all statistical methods, including those used to control for confounding	8	Materials and Methods: section 2.4 Data analysis (paragraph 2-3)
		(*b*) Describe any methods used to examine subgroups and interactions	8	Materials and Methods: section 2.4 Data analysis
		(*c*) Explain how missing data were addressed		Not applicable
		(*d*) *Cohort study*—If applicable, explain how loss to follow-up was addressed<br>*Case-control study*—If applicable, explain how matching of cases and controls was addressed<br>*Cross-sectional study*—If applicable, describe analytical methods taking account of sampling strategy		
		(*e*) Describe any sensitivity analyses		Not applicable
**Results**				
Participants	13*	(a) Report numbers of individuals at each stage of study—eg numbers potentially eligible, examined for eligibility, confirmed eligible, included in the study, completing follow-up, and analysed		Not applicable
		(b) Give reasons for non-participation at each stage		
		(c) Consider use of a flow diagram		
Descriptive data	14*	(a) Give characteristics of study participants (eg demographic, clinical, social) and	8-9	Results (Table 1)
		information on exposures and potential confounders		
		(b) Indicate number of participants with missing data for each variable of interest	8-9	Results (Table 1)
		(c) *Cohort study*—Summarise follow-up time (eg, average and total amount)		Not applicable
		information on exposures and potential confounders		
Outcome data	15*	*Cohort study*—Report numbers of outcome events or summary measures over time		
		Case-control study—Report numbers in each exposure category, or summary measures of exposure		
		Cross-sectional study—Report numbers of outcome events or summary measures	8-14	Results

Main results	16	(*a*) Give unadjusted estimates and, if applicable, confounder-adjusted estimates and their precision (eg, 95% confidence interval). Make clear which confounders were adjusted for and why they were included		Not applicable
		(*b*) Report category boundaries when continuous variables were categorized		Not applicable
		(*c*) If relevant, consider translating estimates of relative risk into absolute risk for a meaningful time period		Not applicable
Other analyses	17	Report other analyses done—eg analyses of subgroups and interactions, and sensitivity analyses	12	Table 7
**Discussion**				
Key results	18	Summarise key results with reference to study objectives	14	Discussion
Limitations	19	Discuss limitations of the study, taking into account sources of potential bias or imprecision. Discuss both direction and magnitude of any potential bias	16	Discussion (last paragraph)
Interpretation	20	Give a cautious overall interpretation of results considering objectives, limitations, multiplicity of analyses, results from similar studies, and other relevant evidence	14-18	Discussion
Generalisability	21	Discuss the generalisability (external validity) of the study results		Not applicable
**Other information**				
Funding	22	Give the source of funding and the role of the funders for the present study and, if applicable, for the original study on which the present article is based	19	Funding

*Give information separately for cases and controls in case-control studies and, if applicable, for exposed and unexposed groups in cohort and cross-sectional studie

Note: An Explanation and Elaboration article discusses each checklist item and gives methodological background and published examples of transparent reporting. The STROBE checklist is best used in conjunction with this article (freely available on the Web sites of PLoS Medicine at http://www.plosmedicine.org/, Annals of Internal Medicine at http://www.annals.org/, and Epidemiology at http://www.epidem.com/). Information on the STROBE Initiative is available at www.strobe-statement.org.
