# Supplementary material for: Cognitive and Behavioral Factors Predicting the Decision to Vaccinate against COVID-19 in Clinical Psychiatric Population—A Cross-Sectional Survey
Source: Vaccines (Basel). 2023 Feb 15;11(2):441. doi: 10.3390/vaccines11020441 (PMC9961952; doi:10.3390/vaccines11020441)
Supplement: Supplementary file 1 [file vaccines-11-00441-s001.zip › Table S2 SM_Normality_tests.pdf]

Table S2: SM_Normality_tests

Descriptives

Descriptives

									Shapiro-Wilk	
	N	Missing	Mean	Median	SD	Variance	Minimum	Maximum	W	p
(1)PERC_RISK_VACC	252	0	3.987	4.286	0.832	0.693	1.00	5.00	0.900	<.001
(1)PERC_RISK_Illness	252	0	5.412	5.500	1.473	2.171	1.00	9.13	0.990	0.076
(2) PERC_VULN	252	0	5.209	5.250	1.732	3.000	1.00	9.25	0.984	0.007
(3)PERC_UNCERTAIN	252	0	6.304	6.500	0.806	0.649	2.00	7.00	0.813	<.001
(4)TRUST_OWN_DEC	252	0	8.190	10.000	2.566	6.585	1	10	0.732	<.001
(6)SOCIAL_MEDIA_BEHAV	252	0	0.992	0.500	1.404	1.970	0.00	10.50	0.691	<.001
(7)PREV_BEHAV	252	0	3.783	3.917	0.674	0.455	1.50	5.00	0.971	<.001

Plots

Legend	
(1) PERC_RISK_VACC	Perceived risk of vaccination
(2) PERC_RISK_VACC	Perceived risk of vaccination
(3) PERC_UNCERTAIN	Perceived uncertainty
(4) TRUST_VACC_DEC	Trust in vaccination decision
(5) PREV_VACC	Previous vaccination
(6) SOCIAL_MEDIA	Social media use
(7) PREV_BEHAV	Engagement in preventive behaviours

(1)PERC_RISK_VACC

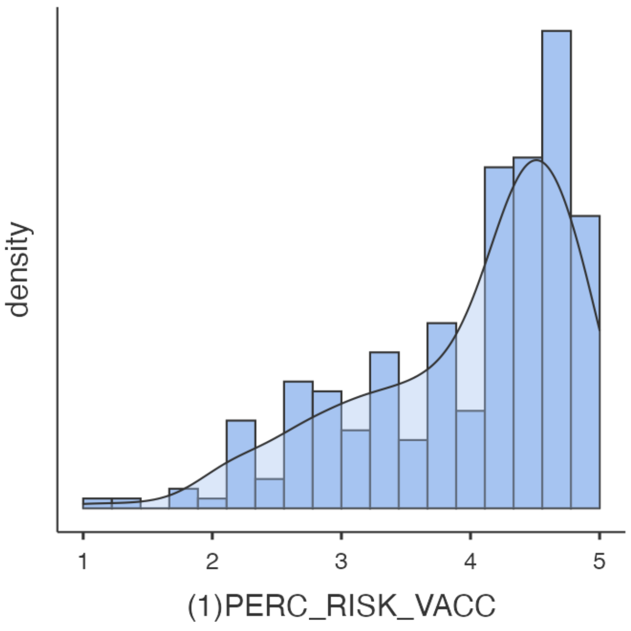

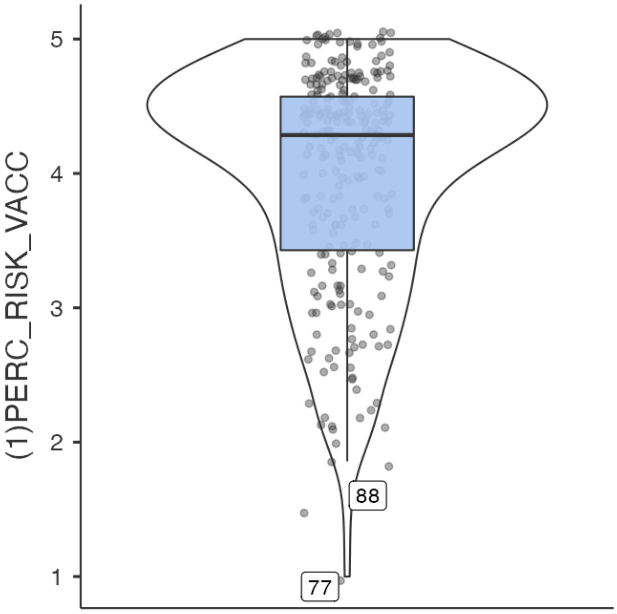

(1)PERC_RISK_Illness

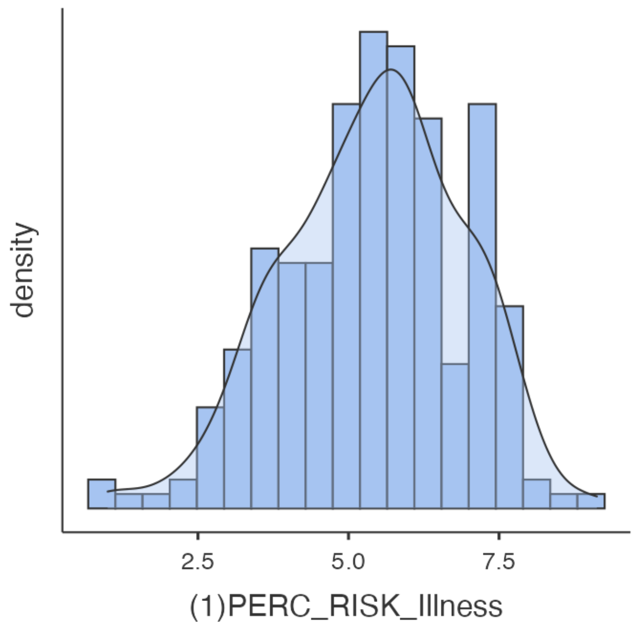

Legend	
(1) PERC_RISK_VACC	Perceived risk of vaccination
(2) PERC_RISK_VACC	Perceived risk of vaccination
(3) PERC_UNCERTAIN	Perceived uncertainty
(4) TRUST_VACC_DEC	Trust in vaccination decision
(5) PREV_VACC	Previous vaccination
(6) SOCIAL_MEDIA	Social media use
(7) PREV_BEHAV	Engagement in preventive behaviours

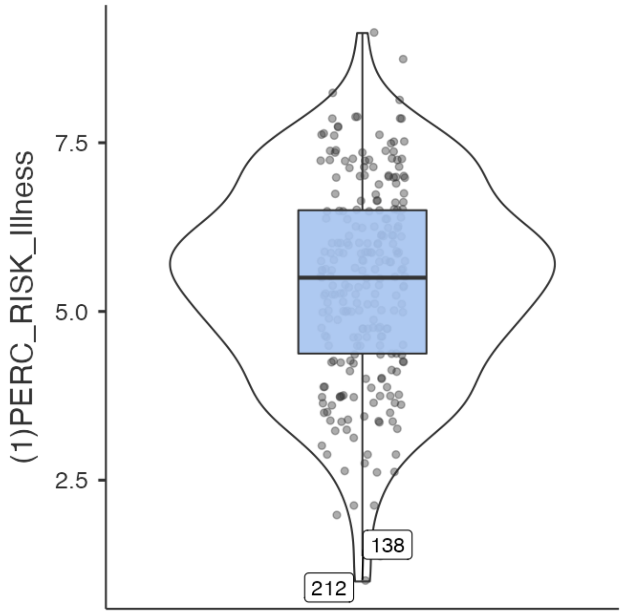

(2) PERC_VULN

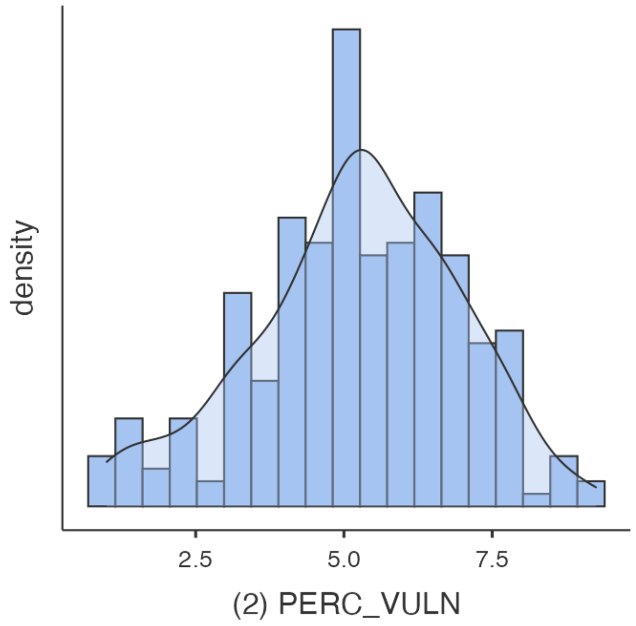

Legend	
(1) PERC_RISK_VACC	Perceived risk of vaccination
(2) PERC_RISK_VACC	Perceived risk of vaccination
(3) PERC_UNCERTAIN	Perceived uncertainty
(4) TRUST_VACC_DEC	Trust in vaccination decision
(5) PREV_VACC	Previous vaccination
(6) SOCIAL_MEDIA	Social media use
(7) PREV_BEHAV	Engagement in preventive behaviours

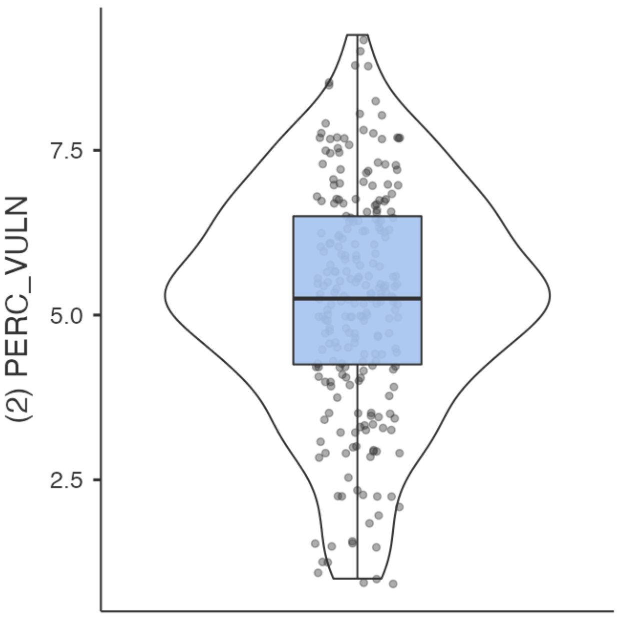

(3)PERC_UNCERTAIN

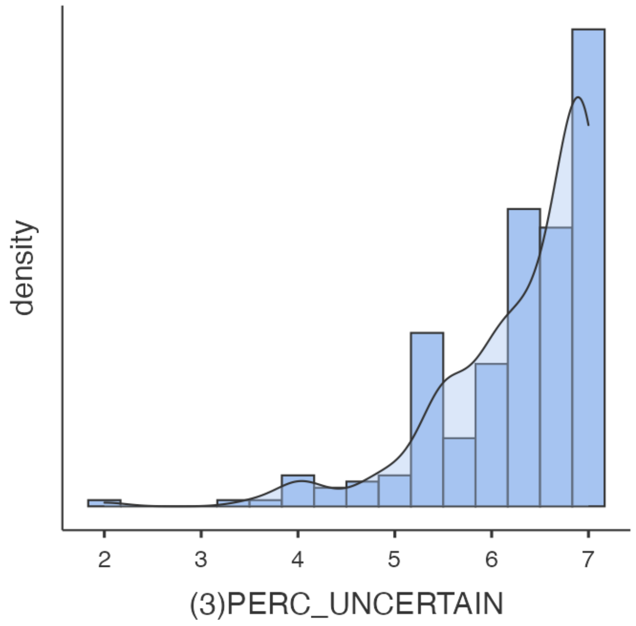

Legend	
(1) PERC_RISK_VACC	Perceived risk of vaccination
(2) PERC_RISK_VACC	Perceived risk of vaccination
(3) PERC_UNCERTAIN	Perceived uncertainty
(4) TRUST_VACC_DEC	Trust in vaccination decision
(5) PREV_VACC	Previous vaccination
(6) SOCIAL_MEDIA	Social media use
(7) PREV_BEHAV	Engagement in preventive behaviours

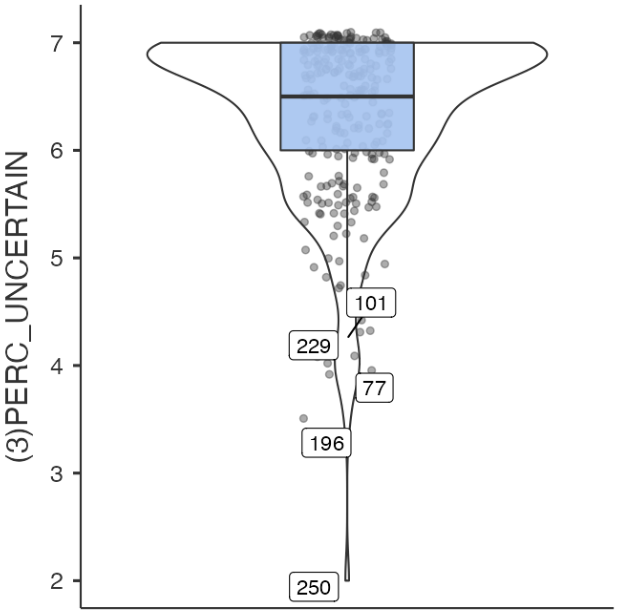

(4)TRUST_OWN_DEC

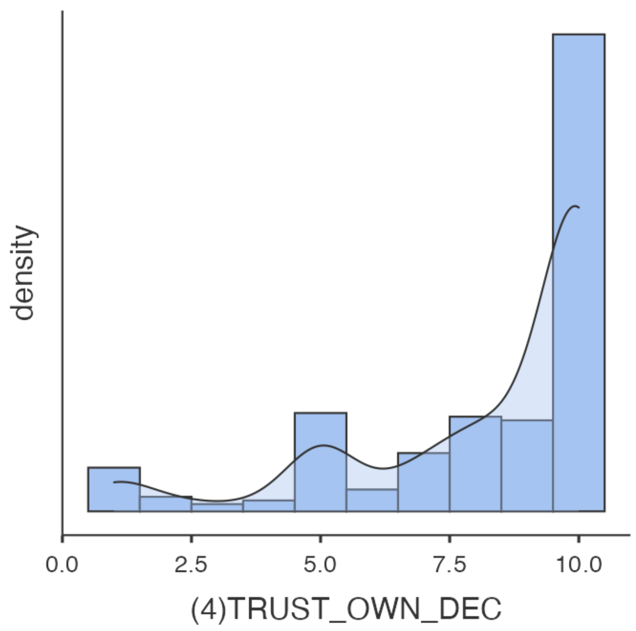

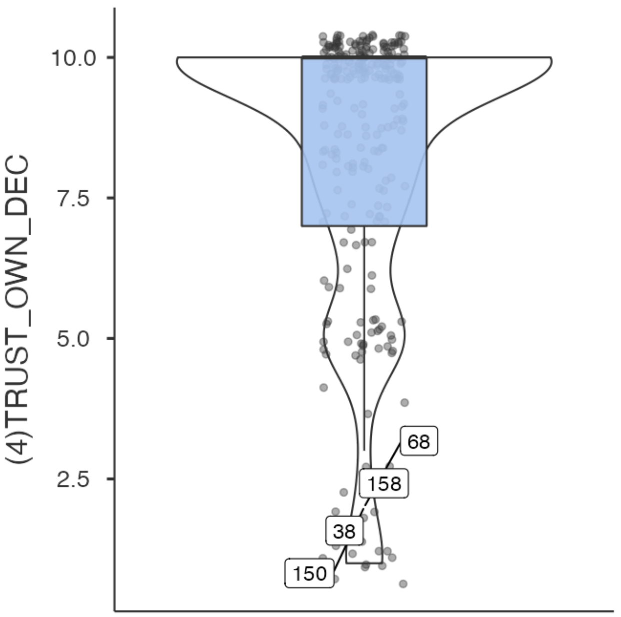

(6)SOCIAL_MEDIA_BEHAV

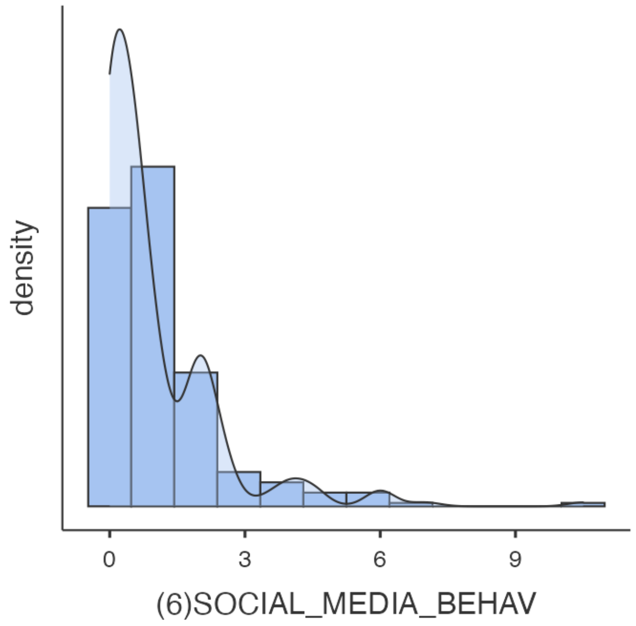
density
0
3
6
9
(6)SOCIAL_MEDIA_BEHAV

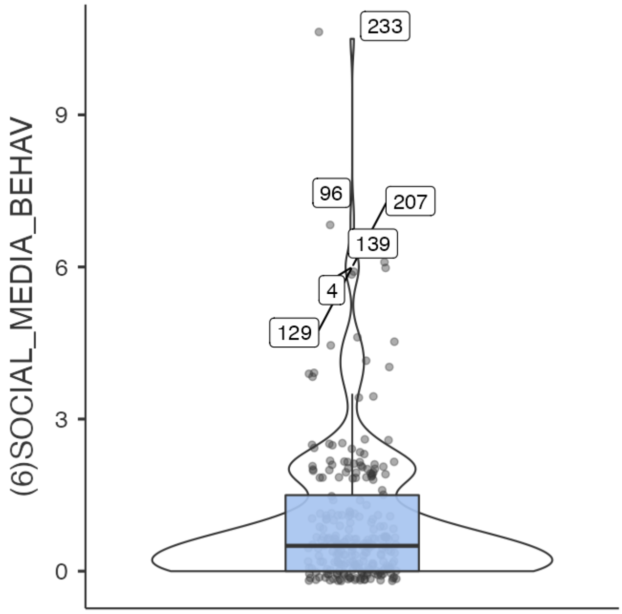
(6)SOCIAL_MEDIA_BEHAV
0
3
6
9
233
96
207
139
4
129

(7)PREV_BEHAV

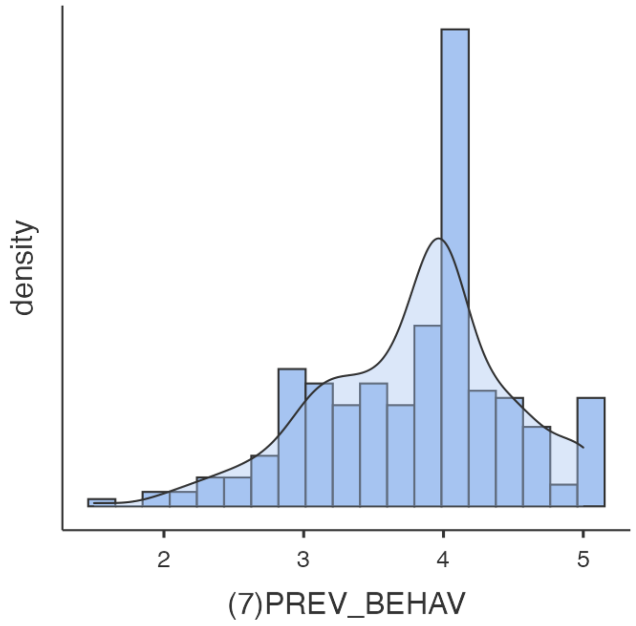
density
2
3
4
5
(7)PREV_BEHAV

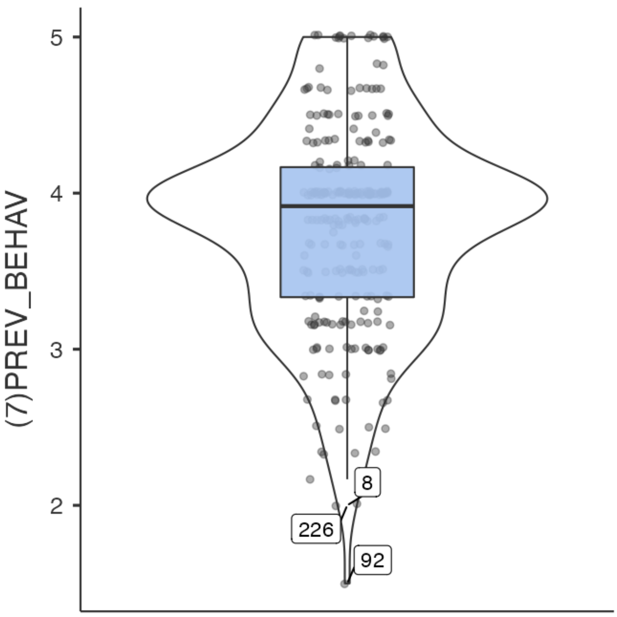
(7)PREV_BEHAV
5
4
3
2
8
226
92
