# Supplementary material for: Cognitive and Behavioral Factors Predicting the Decision to Vaccinate against COVID-19 in Clinical Psychiatric Population—A Cross-Sectional Survey
Source: Vaccines (Basel). 2023 Feb 15;11(2):441. doi: 10.3390/vaccines11020441 (PMC9961952; doi:10.3390/vaccines11020441)
Supplement: Supplementary file 1 [file vaccines-11-00441-s001.zip › Table S3 SM_Logistic_Regression_results.pdf]

| Legend | |
|---|---|
| (1) PERC_RISK_VACC | Perceived risk of vaccination |
| (2) PERC_RISK_VACC | Perceived risk of vaccination |
| (3) PERC_UNCERTAIN | Perceived uncertainty |
| (4) TRUST_VACC_DEC | Trust in vaccination decision |
| (5) PREV_VACC | Previous vaccination |
| (6) SOCIAL_MEDIA | Social media use |
| (7) PREV_BEHAV | Engagement in preventive behaviours |

Table S3: SM_Logistic_Regression_results

# Binomial Logistic Regression

Model Fit Measures

| | | | | | | Overall Model Test | | |
|---|---|---|---|---|---|---|---|---|
| **Model** | **Deviance** | **AIC** | $R^2_{McF}$ | $R^2_{CS}$ | $R^2_N$ | $\chi^2$ | **df** | **p** |
| 1 | 218 | 238 | 0.309 | 0.320 | 0.449 | 97.2 | 9 | < .001 |

Model Coefficients - VACCINAT

| | | | | | | 95% Confidence Interval | |
|---|---|---|---|---|---|---|---|
| **Predictor** | **Estimate** | **SE** | **Z** | **p** | **Odds ratio** | **Lower** | **Upper** |
| Intercept | 0.6092 | 1.980 | 0.308 | 0.758 | 1.839 | 0.0379 | 89.105 |
| (5) PREV_VACC: | | | | | | | |
| Nu – Da | 0.3707 | 0.392 | 0.946 | 0.344 | 1.449 | 0.6722 | 3.123 |
| (4) TRUST_VACC_DEC: | | | | | | | |
| scazut – ridicat | 2.1901 | 0.619 | 3.540 | < .001 | 8.936 | 2.6579 | 30.045 |
| mediu – ridicat | 1.0555 | 0.419 | 2.520 | 0.012 | 2.873 | 1.2645 | 6.529 |
| (1) PERC_RISK_ILL | 0.2333 | 0.146 | 1.600 | 0.110 | 1.263 | 0.9489 | 1.680 |
| (1) PERC_RISK_VACC | -1.5639 | 0.239 | -6.530 | < .001 | 0.209 | 0.1309 | 0.335 |
| (2) PERC_VULN | -0.0309 | 0.120 | -0.257 | 0.797 | 0.970 | 0.7660 | 1.227 |
| (3) PERC_UNCERTAIN | 0.1504 | 0.209 | 0.720 | 0.471 | 1.162 | 0.7719 | 1.750 |
| (6) SOCIAL_MEDIA | -0.0399 | 0.128 | -0.312 | 0.755 | 0.961 | 0.7476 | 1.235 |
| (7) PREV_BEHAV | 0.5170 | 0.273 | 1.895 | 0.058 | 1.677 | 0.9824 | 2.863 |

*Note.* Estimates represent the log odds of "VACCINAT = Nu" vs. "VACCINAT = Da"

## Assumption Checks

Collinearity Statistics

| | **VIF** | **Tolerance** |
|---|---|---|
| (5) PREV_VACC | 1.04 | 0.960 |
| (4) TRUST_VACC_DEC | 1.03 | 0.970 |
| (1) PERC_RISK_ILL | 1.23 | 0.816 |
| (1) PERC_RISK_VACC | 1.08 | 0.929 |
| (2) PERC_VULN | 1.24 | 0.807 |
| (3) PERC_UNCERTAIN | 1.01 | 0.986 |
| (6) SOCIAL_MEDIA | 1.03 | 0.967 |
| (7) PREV_BEHAV | 1.11 | 0.904 |

## Prediction

### Cut-Off Plot

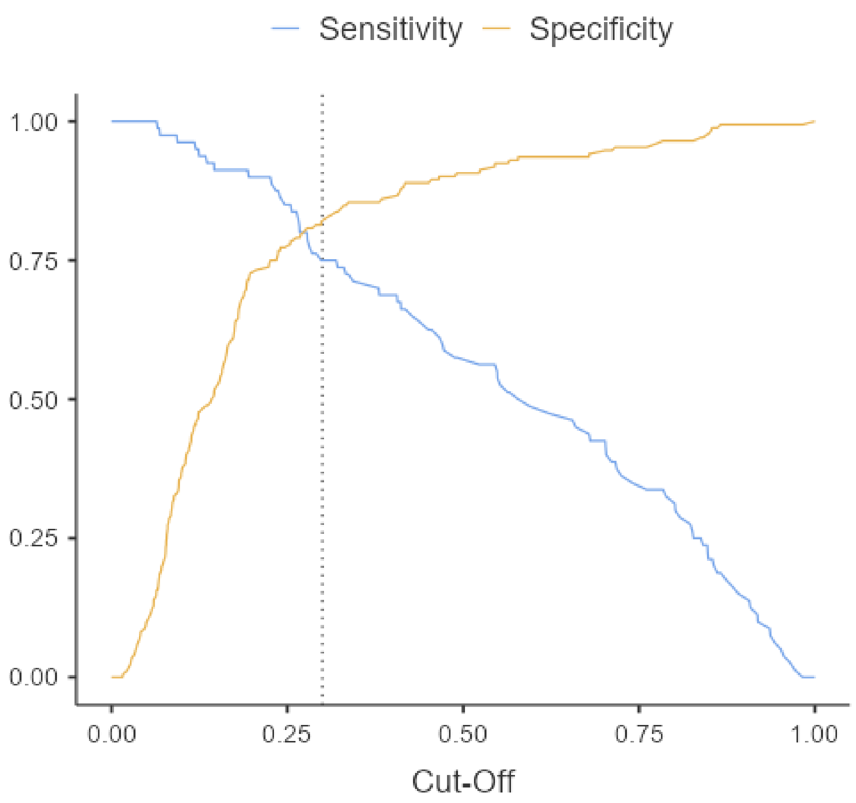


Classification Table – ...

| | Predicted | | |
|---|---|---|---|
| Observed | Da | Nu | % Correct |
| Da | 142 | 30 | 82.6 |
| Nu | 20 | 60 | 75.0 |

*Note*. The cut-off value is set to 0.3

Predictive Measures

| Accuracy | Specificity | Sensitivity | AUC |
|---|---|---|---|
| 0.802 | 0.826 | 0.750 | 0.868 |

*Note*. The cut-off value is set to 0.3

**ROC Curve**

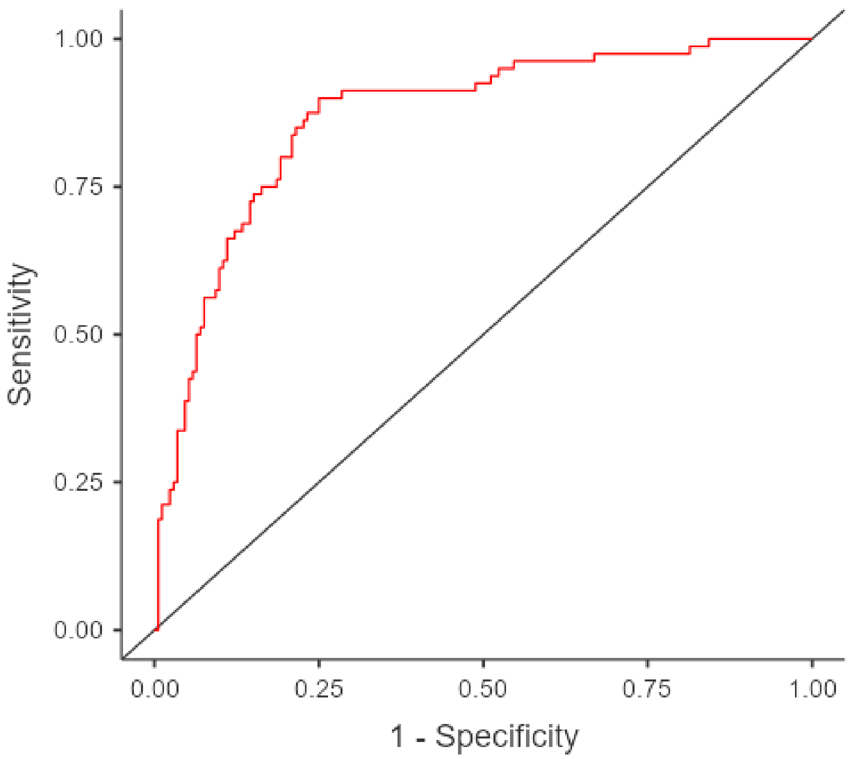

1.00
0.75
0.50
0.25
0.00
Sensitivity
0.00
0.25
0.50
0.75
1.00
1 - Specificity
