# Supplementary material for: Cognitive and Behavioral Factors Predicting the Decision to Vaccinate against COVID-19 in Clinical Psychiatric Population—A Cross-Sectional Survey
Source: Vaccines (Basel). 2023 Feb 15;11(2):441. doi: 10.3390/vaccines11020441 (PMC9961952; doi:10.3390/vaccines11020441)

Table S4: SM_TOST_correlations

TOST Correlation

Legend	
(1) PERC_RISK_VACC	Perceived risk of vaccination
(2) PERC_RISK_VACC	Perceived risk of vaccination
(3) PERC_UNCERTAIN	Perceived uncertainty
(4) TRUST_VACC_DEC	Trust in vaccination decision
(5) PREV_VACC	Previous vaccination
(6) SOCIAL_MEDIA	Social media use
(7) PREV_BEHAV	Engagement in preventive behaviours

```
Correlation method: Spearman's rho

Hypothesis Tested: Equivalence

Bounds: -0.3 - 0.3
```

TOST Results

				90% Confidence Interval			
		r	p	Lower	Upper	Sig. Result	TOST Result
(1)PERC_RISK_VACC	(7)PREV_BEHAV	0.259	<.001	0.157	0.3563	TRUE	FALSE
(2) PERC_VULN	(1)PERC_RISK_VACC	0.211	<.001	0.107	0.3113	TRUE	FALSE
(2) PERC_VULN	(1)PERC_RISK_Illness	0.488	<.001	0.402	0.5657	TRUE	FALSE
(6)SOCIAL_MEDIA_BEHAV	(1)PERC_RISK_Illness	-0.127	0.044	-0.231	-0.0202	TRUE	TRUE
(6)SOCIAL_MEDIA_BEHAV	(1)PERC_RISK_VACC	-0.196	0.002	-0.297	-0.0912	TRUE	TRUE
(6)SOCIAL_MEDIA_BEHAV	(2) PERC_VULN	-0.177	0.005	-0.279	-0.0713	TRUE	TRUE

Descriptives

	N	Mean	Median	SD	SE
(1)PERC_RISK_VACC	252	3.987	4.286	0.832	0.0524
(7)PREV_BEHAV	252	3.78	3.92	0.674	0.0425
(2) PERC_VULN	252	5.209	5.250	1.732	0.1091
(1)PERC_RISK_VACC	252	3.99	4.29	0.832	0.0524
(2) PERC_VULN	252	5.209	5.250	1.732	0.1091
(1)PERC_RISK_Illness	252	5.41	5.50	1.473	0.0928
(6)SOCIAL_MEDIA_BEHAV	252	0.992	0.500	1.404	0.0884
(1)PERC_RISK_Illness	252	5.41	5.50	1.473	0.0928
(6)SOCIAL_MEDIA_BEHAV	252	0.992	0.500	1.404	0.0884
(1)PERC_RISK_VACC	252	3.99	4.29	0.832	0.0524
(6)SOCIAL_MEDIA_BEHAV	252	0.992	0.500	1.404	0.0884
(2) PERC_VULN	252	5.21	5.25	1.732	0.1091

Plots

(1)PERC_RISK_VACC - (7)PREV_BEHAV

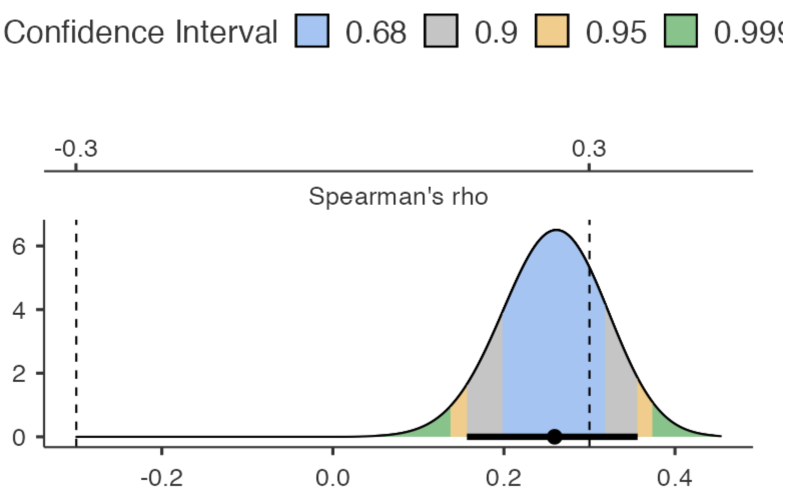

(2) PERC_VULN - (1)PERC_RISK_VACC

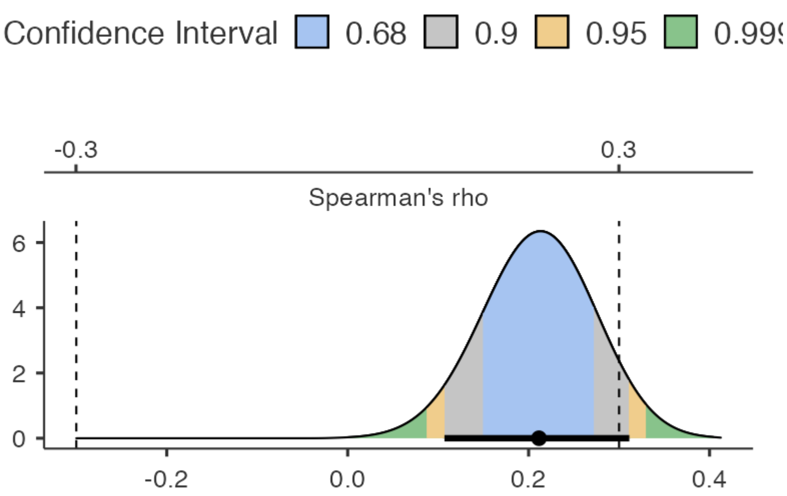

(2) PERC_VULN - (1)PERC_RISK_Illness

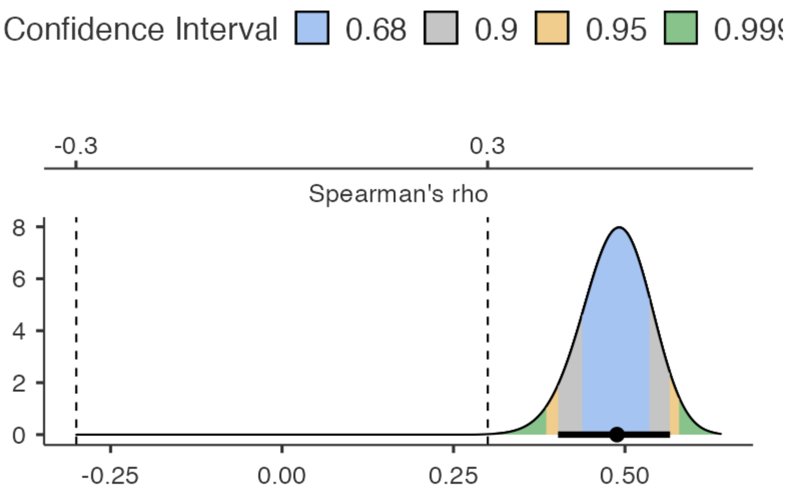

(6)SOCIAL_MEDIA_BEHAV - (1)PERC_RISK_Illness

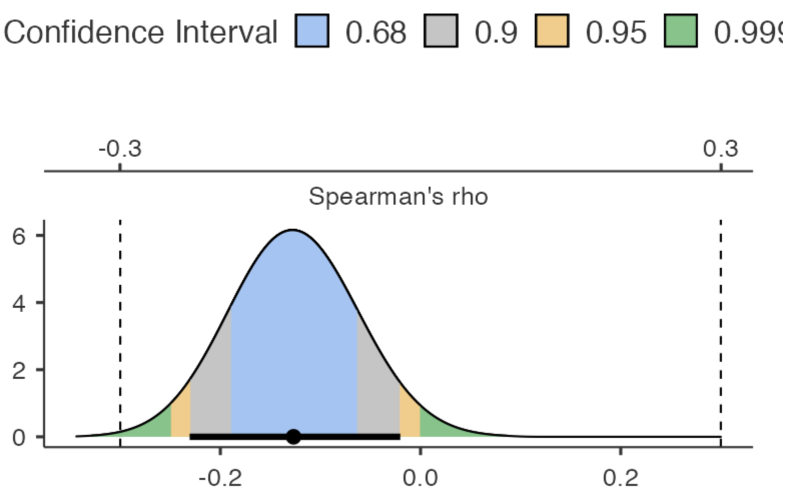

(6)SOCIAL_MEDIA_BEHAV - (1)PERC_RISK_VACC

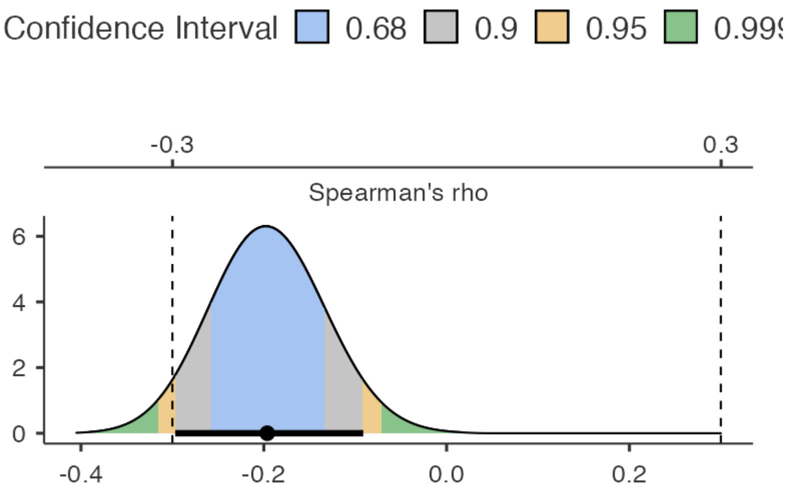

(6)SOCIAL_MEDIA_BEHAV - (2) PERC_VULN

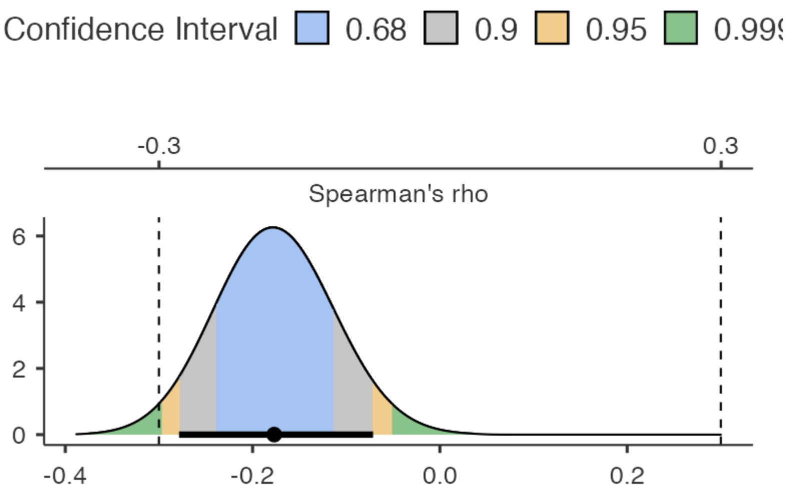

Supplement: Supplementary file 1 [file vaccines-11-00441-s001.zip › Table S4 SM_TOST_correlations.pdf]
